# Cytoplasmic Domain of MscS Interacts with Cell Division Protein FtsZ: A Possible Non-Channel Function of the Mechanosensitive Channel in *Escherichia Coli*

**DOI:** 10.1371/journal.pone.0127029

**Published:** 2015-05-21

**Authors:** Piotr Koprowski, Wojciech Grajkowski, Marcin Balcerzak, Iwona Filipiuk, Hanna Fabczak, Andrzej Kubalski

**Affiliations:** Department of Cell Biology, Nencki Institute of Experimental Biology, Polish Academy of Sciences, Pasteur 3, Warsaw, Poland; University of Bern, SWITZERLAND

## Abstract

Bacterial mechano-sensitive (MS) channels reside in the inner membrane and are considered to act as emergency valves whose role is to lower cell turgor when bacteria enter hypo-osmotic environments. However, there is emerging evidence that members of the **M**echano-**s**ensitive **c**hannel **S**mall (MscS) family play additional roles in bacterial and plant cell physiology. MscS has a large cytoplasmic C-terminal region that changes its shape upon activation and inactivation of the channel. Our pull-down and co-sedimentation assays show that this domain interacts with FtsZ, a bacterial tubulin-like protein. We identify point mutations in the MscS C-terminal domain that reduce binding to FtsZ and show that bacteria expressing these mutants are compromised in growth on sublethal concentrations of β-lactam antibiotics. Our results suggest that interaction between MscS and FtsZ could occur upon inactivation and/or opening of the channel and could be important for the bacterial cell response against sustained stress upon stationary phase and in the presence of β-lactam antibiotics.

## Introduction

The physiological function that is ascribed to mechanosensitive (MS) channels in bacteria is that they jettison osmolytes and, thus, play an important role in the survival of severe osmotic downshocks [1,2]. In *E*. *coli* the pentameric large-conductance MscL and the heptameric small-conductance MscS, both residing in the inner membrane, are the best characterized. These channels operate over distinct ranges of membrane tension, with MscS opening when a cell begins to swell as a result of water influx, and MscL opening at near-lytic tensions [3]. Both channels are activated directly by membrane stretch with no cytoskeletal elements required for their activation [4].

**Fig 1:**
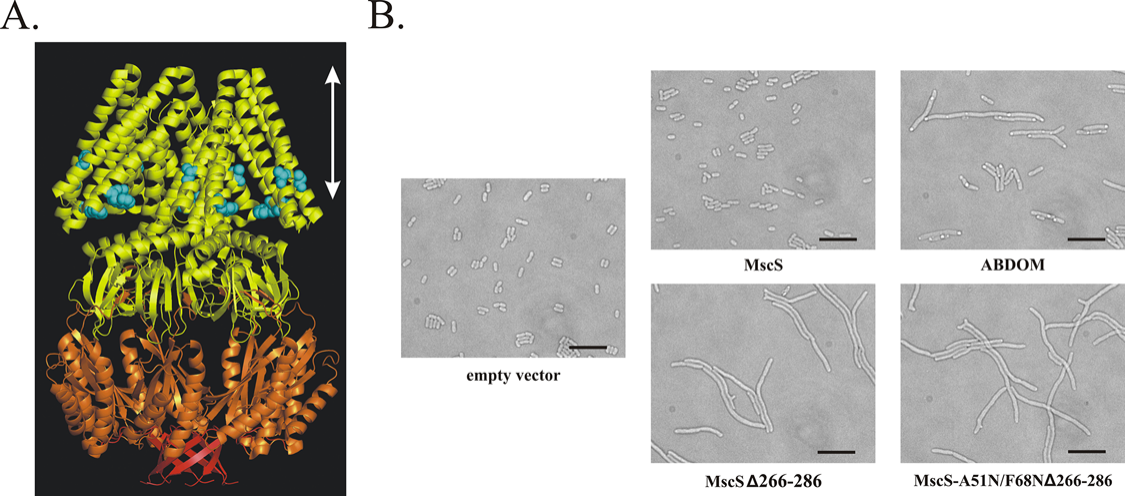
MscS, the structure and its mutations affecting cell shape. **A.** Crystal structure of the MscS homoheptamer (PDB ID:2OAR) representing the nonconductive state of the channel [36]. ABDOM (aa 175–265) of each subunit is shown in orange and the C-terminal fragment (aa 266–286), which is deleted in the MscSΔ266–286 mutant, is shown in red. White arrow indicates the thickness of the membrane. **B.** Overexpression of **ABDOM** leads to cell filamentation, while cells expressing **wt-MscS** did not differ in shape from control cells (**empty vector**). The highest level of cell filamentation was observed when truncated **MscSΔ266–286** was expressed (note the presence of branched filaments). The amount of regular cell filaments induced by MscSΔ266–286 was unaffected by double mutation **A51N/F68N**, which prevents ion conduction through the channel pore. Scale bar represents 10 μm. Expression of the proteins was confirmed by Western blotting variants tagged with HA epitope (S1 Fig).

MscL has relatively simple closed—open—closed transitions during gating [5]. In contrast, after opening, the MscS channel undergoes inactivation that completely shuts the channel under sustained force [6,7]. As patch-clamp experiments show, a release of tension is a prerequisite for a return transition from the inactivated to the closed state for MscS [7,8]. Important insight into the molecular mechanism of gating has emerged from crystal structures of MscS, which have provided information on the inactivated [9] and partially open [10] conformations, as well as from kinetic analysis, electron paramagnetic resonance (EPR) studies and computational studies (reviewed in [11]). While these works led to an understanding of the conformational changes of the transmembrane domains and the force transmission between lipids and the channel, as well as solute transport through the gate, less is known about the large cytoplasmic (aa 133–286) region of MscS [9] (Fig 1, panel A). The cytoplasmic part of MscS forms a structure resembling a chamber with seven openings at the side and one at the bottom. The chamber is believed to serve as a molecular sieve [9,12,13], however, it does not form a rigid structure, as one might expect of a sieve. Instead, as demonstrated by experiments and molecular dynamics simulations, the chamber undergoes large conformational changes during channel activation, inactivation and closing, which change its shape and volume [14–20].

We demonstrate here that a cytoplasmic fragment *of E*. *coli* MscS, the **α**/**β D**omain **o**f **M**scS (ABDOM) (residues 175–265, Fig 1, panel A), interacts with FtsZ, a bacterial homolog of tubulin, which has a role in the formation of the Z-ring that initiates cell division [21,22]. We demonstrate that overexpression of the soluble ABDOM disrupts cell division resulting in elongated (filamentous) cells. A similar effect is induced by overexpression of a deletion mutant MscSΔ266–286 with the last 20 amino acids removed (a deletion resulting in a channel that may eventually stay permanently inactivated [15]) but not by the overexpression of wild type (wt) MscS.

We show here that overexpression of MscS protects cells cultured in the presence of subminimal inhibitory concentrations (subMIC) of the β-lactam antibiotic ampicillin. We found also mutations in the ABDOM that reduce its binding to FtsZ and diminish the ability of MscS to protect cells from subMIC ampicillin. Based on these results, we hypothesize that the exposed ABDOM (upon channel opening and inactivation [17]) binds FtsZ and modulates FtsZ-dependent processes including cell wall synthesis and its repair.

## Materials and Methods

### Strains and plasmids

*E*. *coli* strains Frag1, MJF429*ΔyggB ΔkefA*::*kan* and MJF465 *ΔyggB ΔkefA*::*kan ΔmscL*::*cm* were kindly provided by I. R. Booth (University of Aberdeen, Aberdeen, UK). Frag1 is a wt strain, a derivative of *E*. *coli* K-12. *E*. *coli* strains BW25113 *F*, *Δ(araD-araB)567*, *ΔlacZ4787(*::*rrnB-3)*, *λ* ^*-*^, *rph-1*, *Δ(rhaD-rhaB)568* and isogenic strain JW2891-2 carrying deletion of *mscS* gen (*ΔmscS775*::*kan*) were obtained in *E*. *coli* Genetic Stock Center (http://cgsc.biology.yale.edu/). MscS alleles were expressed in MJF429 throughout the work unless stated otherwise.

### Construction of expression vectors

pTRC-*mscS* construct is *mscS* ORF coding for wild-type MscS in pTRC99a vector. It was constructed by making STOP mutation in position 287 in the previously described construct pYggB-His_6_. The primers used were:

5’-GGTGAAAGAAGACAAAGCTGCGTAACACCATCACCATCACTAAGG-3’ and

5’-CCTTAGTGATGGTGATGGTGTTACGCAGCTTTGTCTTCTTTCACC-3’. Basing on this construct the following constructs were made by site-directed mutagenesis. pTRC-*mscSΔ266–286* has STOP mutation in position 266 (primers used:

5’-GAATTTGATGCCGCCGGTTAAAGCTTCCCGTACCCGC-3’ and

5’-GCGGGTACGGGAAGCTTTAACCGGCGGCATCAAATTC-3’).

pTRC-*mscS51N*,*68N* has A51N mutation (primers used:

5’-CGCGCGGATGATTTCCAACAACGTGAATCGCCTGATGATCTC-3’ and

5’-GAGATCATCAGGCGATTCACGTTGTTGGAAATCATCCGCGCG-3’)

and F68N mutation (primers used:

5’-ATCGATGCCACTGTTGCTGATAATCTTTCTGCATTAGTCCG-3’ and

5’-CGGACTAATGCAGAAAGATTATCAGCAACAGTGGCATCGAT-3’).

pTRC-*mscS51N*,*68NΔ266–286* has all the three mutations: F266STOP, A51N and F68N. All mutation reactions were performed using QuikChange Site-Directed Mutagenesis Kit (Stratagene).

pTRC-*abdom* construct was created by amplifying DNA fragment coding for the ABDOM domain of MscS (aa form I175 to S261, with MAF fragment added N-terminally) with pTRC-*mscS* as the template (primers used:

5’-TTACCATGGAATTCATTATTAACTTCTCCCGCGA-3’ and

5’-GCCTAGGTTAGCTGATACCGGCGGCA-3’)

and cloning the product in pTRC99a vector in *EcoR*I-*BamH*I. pGEX-*abdom* construct for GST-ABDOM expression was made by amplifying the fragment coding for ABDOM domain with pTRC-*mscS* as the template (primers used:

5’-TTACCATGGAATTCATTATTAACTTCTCCCGCGA-3’ and

5’-AGTCGCGGCCGCTTAGCTGATACCGGCGGCATCAAATTC-3’)

and cloning the product in pGEX4T-1 in *EcoR*I-*Not*I. pMAL-*abdom* construct for MBP-ABDOM expression was made by amplifying the fragment coding for ABDOM domain with pTRC-*mscS* as the template with primers

5’-.ATTGAATTCTCCCGCGAGCCAGTTCGCCGTAAC-3’ and

5’-CGGGGATCCTTAGCTGATACCGGCGGCATCAAATTCAC-3’

and cloning the product in pMAL-C2E vector in *EcoR*I-BamHI.

Both pET11-*ftsZ* and pJSB2-*ftsZYF* plasmids were kind gifts from Prof H. P. Erickson (Duke University Medical Center, Durham, NC).

pBAD-*ftsZ* construct was made by amplifying *ftsZ* gene with pET11-*ftsZ* as the template (primers used:

5’-TAGAGCTCATGTTTGAACCAATGGAACTTACC-3' and

5’-ATAAGCTTAATCAGCTTGCTTACGCAGG-3')

and cloning the PCR product in pBAD33 in *Sac*I-*Hind*III.

### GST pull-down assay

The GST-ABDOM fusion protein was expressed in MJF429 strain at a very low level (uninduced promoter). The GST-ABDOM fusion protein was purified on Glutathione Sepharose 4B (GE Healthcare) according to the manufacture instructions with modifications The cells where harvested by centrifugation, suspended in the binding buffer containing: PBS pH 7.5, 1mM MgCl_2_, 10% glycerol, 3mM PMSF, 10l/ml Protease Inhibitor Cocktail (Sigma), lysozyme 1mg/ml, DNase 1mg/ml and sonicated. Triton X-100 was added to concentration 0.5% and the lysate was cleared by centrifugation at 21,000 x g at 4^°^C. The supernatant was incubated with Glutathione Sepharose 4B beads (Amersham Biosciences) for 25 minutes at 23^°^C. A column was formed and the beads were washed with 30 bead volumes of binding buffer with 0.5% Triton X-100 and inhibitors followed by 15 bead volumes of binding buffer with 0.5% Triton X-100. The fusion protein was eluted with glutathione elution buffer (100mM Tris-HCl pH 8.0, 125mM NaCl, 3 mg/ml glutathione). The fusion protein was concentrated with Microcon Centrifugal Filter Device with NMWL 3,000 Da (Millipore). Identical procedure was applied to cells expressing GST as a control experiment. Eluates were analyzed by mass spectrometry.

### Protein expression and purification

*E*. *coli* FtsZ was purified as described previously [23], using GTP-precipitation method. BL21(DE3) cells carrying pET11-FtsZ vector were disrupted using a French press (Thermo Scientific) at 120,000 psi. Lysate was centrifuged at 100,000 x g for 1h at 4°C. GTP induced precipitation was performed on supernatant and repeated four times. The Q-sepharose column step was omitted. Final FtsZ pellet was suspended in 50 mM PIPES, pH 6.5, 1 mM EGTA, 5 mM MgCl_2_ at concentration of 10 mg/ml.

ABDOM domain we expressed and purified it with N-terminally fused maltose binding protein (MBP) using amylose resin (Pharmacia) column chromatography. Maltose eluate was dialyzed against 20 mM Tris pH 7.4, 1 mM EGTA. MBP protein for control experiments was also purified as described above. The protein concentration was determined using a Biorad Protein Assay (BioRad). SDS-PAGE analysis of purified proteins is shown in S2 Fig.

### Light scattering

FtsZ polymerization was monitored by 90° angle light scattering in a Fluorog 3 fluorometer (Jobin Yvon Spex) with both the excitation and emission wavelengths set at 350 nm. FtsZ (12 μM) alone or in the presence of MBP-ABDOM (12 μM) in buffer containing (in mM) 50 PIPES pH 6.0, 1 EDTA, 5 MgCl_2_ was added to fluorometer cuvette with a 5-mm path length placed in a thermostated at 30°C chamber equipped with a magnetic stirrer. FtsZ polymerization was induced by addition of 1 mM GTP (Sigma) under constant stirring. Data points were collected every second and plotted with the Microcal Origin 4.1 software.

### Co-sedimentation assay

FtsZ (0–40 μM) was incubated with 8 μM MBP-ABDOM in 50 mM PIPES, pH 6.5, 1 mM EDTA, 5 mM MgCl_2_, 5mM CaCl_2_ for 5 min. Then, 2 mM GTP was added to reaction mixture and incubated at 30°C for 1 min. Samples were centrifuged at 250,000 x g (Sorvall Discovery M120). Equal volumes of suspended pellets were separated on SDS-PAGE and Western blotted with monoclonal mouse anti-MBP antibody (Abcam).

### Microscopy

Aliquots of 10μl of cell culture were dropped on 1% agarose pads made on glass slides, left to dry and covered with coverslips. The images where obtained using Olympus IX70 inverted microscope with Olympus UPlanSApo 60x/1.20 lens (water immersion) and CCD camera Sensicam 12BIT (PCO CCD Imaging). All images were analyzed with the Adobe Photoshop 7.0 software.

For cell filamentation assay depicted in Fig 1, MJF429 cells carrying different constructs (all being pTRC99a derivatives) were induced with 1 mM IPTG at OD_600_ of 0,1 and grown at 37^°^C and 200rpm shaking. The images were taken 3 h after the induction. At this point OD_600_ of the three cultures of filamentous cells was about 0.9 compared to 1.2 in case of the rest of cultures.

For fluorescence microscopy cells of MJF429 strain carrying pJSB2-*ftsZYFP* plasmid [24] and either pTRC-*mscSΔ266–286* or pTRC99A were diluted from overnight culture and grown in LB media with chloramphenicol and ampicillin to OD_600_ of 0.1. Subsequently arabinose and IPTG were added to 0.0002% and 1mM, respectively. Cells were grown at 37^°^C and 200 rpm shaking and images where taken 1 h after the induction. Cells were fixed with 2.5% paraformaldehyde in PBS for 15 min at room temperature and 45 min on ice.

### Growth in ampicillin

MJF429 strain carrying pWG1 vector (kanR derivative of pTRC99A) alone, or expressing MscS, or MscS-K258A/R259A, or MscS-YFP was grown in LB170 (control) or in LB170 with 1.6 μg/ml or 4.1μg/ml ampicillin.

### D-cysteine labeling and immunolabelling of peptidoglycan sacculi

MJF429 cell cultures carring pTRC-MscSΔ(268–286) or pTRC-ABDOM vectors grown overnight were diluted in LB broth containing 100 μg/ml ampicillin. After 10 min 200μg/ml D-cysteine (USBiological) was added and incubated for 30 min at 37°C. Then, 1 mM IPTG was added. After 1h incubation cells were collected by centrifugation (4 min, 5,000 x g) and resuspended in D-cysteine-free medium prewarmed to 37° and incubated for additional 30 min. Then, cells were centrifuged for 4 min at 5,000 x g and suspended in 0.9% NaCl. Sacculi were isolated and D-cysteine residues were biotinylated using (*N*-(6-(Biotinamido)hexyl)-3'-(2'-pyridyldithio)-propionamide (Pierce) as described previously (de Pedro et al., 1997). Biotin was detected using monoclonal mouse anti-biotin antibody conjugated with Alexa Fluor 488 (Invitrogen) diluted 1:300 into 0.2% gelatin and 0.5% BSA in PBS, pH 7.3.

### Mass spectrometry

There were two independent GST pull-down experiments performed. In the first one the concentrated eluates were directly subjected to standard trypsin digestion, while in the second, the eluted proteins were first separated on 12% SDS-PAGE gel. The gel was silver-stained, the bands were excised and in-gel trypsin digestion was performed. The peptide mixtures obtained in each experiments was applied to a RP-18 precolumn (LC Packings) using water containing 0.1% trifluoroacetic acid as a mobile phase and then transferred to a nano-HPLC RP-18 column (LC Packings, 75 μM i.d.) using an acetonitrile gradient (0%- 50% acetonitrile in 30 minutes) in the presence of 0.05% formic acid at a flow rate of 150 nl/min. The column outlet was directly coupled to an ion source of the LTQ FTICR spectrometer (Thermo) working in the regime of data dependent MS to MS/MS switch.

The mass spectra obtained in both experiments were used to search the non-redundant protein database of National Center of Biotechnology Information (NCBI) using the MASCOT search engine (Matrixscience.com). Search parameters were set as follows: taxonomy restriction—all entries, enzyme—trypsin; fixed modification—carbamidomethylation (C); variable modifications—carbamidomethylation (K), oxidation (M); protein mass—unrestricted; peptide mass tolerance +/- 40 ppm; fragment mass tolerance—+/-0.8 Da; max missed cleavages—1. Only protein hits characterized by the presence of at least one peptide with a score > 75 were accepted. All peptide hits with score > 75 were also validated by manual inspection of the MS/MS data. Hits were accepted if all strong peaks in the MS/MS spectrum were assigned either to y- or b- series ions and the spectrum contained y- or b- series signals corresponding to at least one stretch of three or more consecutive amino acids.

### Flow cytometry

Cells were grown in LB supplemented with kanamycin and with or without ampicillin. For flow cytometry measurements (FASCalibur, BD Biosciences) cell cultures were diluted in the same media if needed. For cell death determination cells were incubated for 10 min before measurement in 10μg/ml propidium iodide (PI). PI fluorescence was measured with Ex533nm/>Em670nm.

Data were presented as means *±* SD of three experiments.

### Patch clamp

All patch-clamp experiments were done as previously described [19]. Pressure to the recording pipette was applied manually or by the high speed pressure clamp (HSPC-1, ALA Scientific Intruments). Purified proteins, FtsZ or MBP were added to 16 μM.

## Results

### Cell filamentation is induced by expression of either the isolated ABDOM or of MscSΔ266–286 mutant

In our previous work we demonstrated that the MscS cytoplasmic chamber changes its shape upon channel kinetic transitions [17]. We wanted to know if the movement of its cytoplasmic domains is eventually involved in an interaction with other proteins. We focused on the ABDOM (residues 175–265) and the region that follows immediately after (Fig 1, panel A).

Overexpression of the 90-residues-long ABDOM in *E*. *coli* induced a striking cell filamentation (Fig 1, panel B). We asked whether a filamentation would also be induced by overexpression of the full-length MscS channel and we found that it was not the case (Fig 1, panel B). We wondered whether this lack of effect was due to the membrane-associated localization of the ABDOM in the intact channel, or to a limitation of its exposure to possible interaction partners. We addressed this question using a truncated mutant MscSΔ266–286, from which the last 20 amino acids of the C-terminus immediately following the ABDOM were missing. These amino acids form a separate ß-barrel structure and its deletion shouldn’t affect the structure of ABDOM. MscSΔ266–286 mutant opens at slightly higher threshold and inactivates normally, but has defective recovery from inactivation [15], indicating that it stabilizes a non-conducting, possibly inactivated-like state. Nevertheless, overexpressed MscSΔ266–286 protects cells against hypo-osmotic shock, indicating that the channel is physiologically functional but the configuration of its cytoplasmic domain is different from the full-length MscS [15]. We found that overexpression of the MscSΔ266–286 mutant channel resulted in cell filamentation that was similar or stronger to what we saw in cells overexpressing the ABDOM alone (Fig 1, panel B).

To be sure that the conduction of solutes through the channel was not responsible for the cell filamentation we introduced a loss-off-function mutation A51N/F68N [25] into the transmembrane domain of the MscSΔ266–286 mutant. In both the wild-type background and the MscSΔ266–286 background introduction of the A51N/F68N mutations eliminated the channel’s ability to protect cells from osmotic down-shocks, consistently with their disability to conduct solutes (not shown). Nevertheless, the impaired version of MscSΔ266–286 was fully proficient in inducing cell filamentation (Fig 1, panel B). This result demonstrates that current flow through the channel was *not* responsible for cell filamentation. Instead, the simplest explanation appears to be that the MscSΔ266–286 mutation stabilized a conformation of the channel that exposed the ABDOM and thus mimicked the effect of expression of ABDOM alone. However, the effect on cell length suggests that ABDOM may interact with regulators of bacterial cell shape and/or cell division.

### ABDOM interacts with FtsZ

In an attempt to identify possible interaction partners of ABDOM, we carried out a pull-down assay from bacterial lysates followed by mass spectrometry, using the ABDOM fused to glutathione-S-transferase (GST) as the bait. To avoid non-specific protein interactions within the cell, the GST-ABDOM fusion was expressed at low level, at which no cell filamentation was observed. In two independent experiments we identified several proteins that bound to the GST-ABDOM fusion, but not to GST alone. These were FtsZ, ClpX, OmpC, RecA, HCP, and MreB (S1 Table). Several of these proteins are found together in complexes that probably regulate cell division and cell shape [26]. Among the ABDOM-interacting proteins we selected FtsZ for further investigation for the following reasons: i. it was identified with the highest confidence score; ii. we expected that excessive ABDOM binding may interfere with a primary function of the partner and that, therefore, its expression effect on cell filamentation could be accounted for by a block of FtsZ function [21]; iii) ClpX interacts with FtsZ [27,28] suggesting that its binding to ABDOM may be indirect (i.e. mediated through FtsZ), and, moreover, we observed the ABDOM-induced filamentation in *clpX* ^*—*^strain (data not shown); iv. we observed the ABDOM-induced filamentation in *recA* ^*—*^strain (data not shown); v. MreB was identified with a low score and interference with its function results in round cells and not in the cell filamentation [29]. Moreover, MreB possibly associates with FtsZ [26], so that its interaction with ABDOM could be indirect.

**Fig 2:**
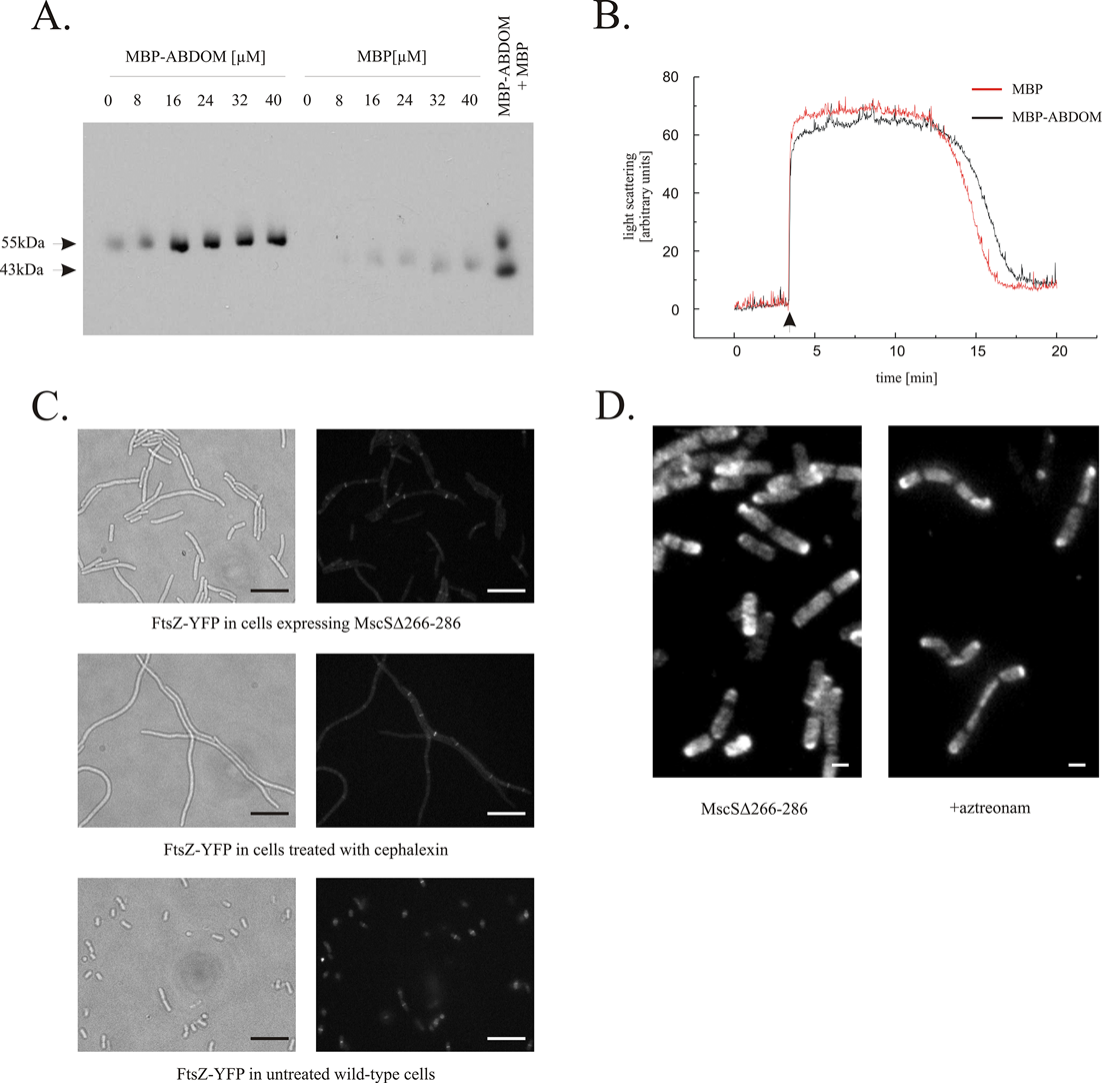
ABDOM binds FtsZ but does not interfere with its polymerization neither *in vitro* nor *in vivo*. A. Purified MBP-ABDOM bound assembled FtsZ *in vitro*. The MBP-ABDOM fusion protein, but not MBP alone, co-sedimented with polymerized FtsZ. Fixed concentration of FtsZ (16 μM) was co-sedimented by addition of 1mM GTP in the presence of increasing concentration of MBP or MBP-ABDOM. For reference, equal amounts of MBP-ABDOM and MBP supernatant without FtsZ are shown in rightmost lane. Proteins were detected with anti-MBP antibody. **B.** MBP-ABDOM had no effect on FtsZ polymerization/depolymerization *in vitro*, as detected by 90° angle light scattering. FtsZ was polymerized after addition of 1 mM GTP (arrow) in the presence of either MBP-ABDOM (black line) or MBP (red line) **C.** Filaments produced by MscSΔ266–286 expression contain multiple nonfunctional Z-rings (upper row), which resemble Z-rings arrested by the cephalexin inhibition of PBP3 (middle row), control cells (lower row). FtsZ-YFP was expressed to visualize Z-rings. Scale bar represents 10 μm. **D.** Peptidoglycan synthesis detected by immunofluorescent D-cysteine labeling. Overexpression of MscSΔ266–286 resulted in cell filamentation, with clear dark rings of newly synthesized murein in septal areas (left). A similar pattern of murein segregation was observed in cells treated with 1μg/ml aztreonam, a selective inhibitor of PBP3 whose septal localization depends on functional Z-rings (right). Experiments were carried out over one cell cycle in D-cysteine, followed by 30 min. chase. The labeled, older murein is seen as bright spots, while newly made murein is seen as dark areas. Scale bar represents 1 μm.

To determine if FtsZ interacts with ABDOM directly, we assayed co-sedimentation of FtsZ with a fusion of ABDOM to maltose binding protein (MBP). In contrast to dimeric GST, MBP is monomeric, therefore advantageous in co-sedimentation experiments. MBP-ABDOM, MBP and FtsZ were each purified (S2 Fig), combined in a pair-wise fashion and tested *in vitro*. We found that MBP-ABDOM, but not MBP alone, co-sedimented with GTP-polymerized FtsZ (Fig 2, panel A), indicating that ABDOM interacts directly with a polymerized form of FtsZ. No co-sedimention of MBP-ABDOM was observed in the absence of either GTP (S3 Fig, panel B) or FtsZ (S3 Fig, panels A and C).

Proteins that inhibit FtsZ polymerization induce cell filamentation when over-expressed [30]. Knowing that ABDOM expression results in cell filamentation, we wondered if ABDOM binding influences FtsZ polymerization. FtsZ polymerizes into long protofilaments in a GTP-dependent manner. This polymerization can be monitored by 90° angle light scattering [31]. Proteins that inhibit FtsZ polymerization decrease light scattering [32]. We found that light scattering was not affected when MBP-ABDOM was added to media containing FtsZ and GTP, indicating that neither polymerization nor depolymerization of FtsZ is favored *in vitro* (Fig 2B). This indicates that ABDOM is not an inhibitor of FtsZ polymerization.

### Z-ring formation and murein synthesis in cells overexpressing MscSΔ266–286 mutant

Overexpression of some proteins that interact with FtsZ impair the formation of Z-rings or cause Z-ring dysfunction, thereby leading to cell filamentation [33]. We observed that Z-rings were formed in cell filaments that were induced by MscSΔ266–286 overexpression (Fig 2, panel C) and we found that their number and the distances between them appeared to be similar to those found in cell filaments induced by cephalexin treatment. Cephalexin blocks activity of penicillin binding protein 3 (PBP3) which acts downstream of FtsZ and thereby does not interfere with Z-ring formation. Our observation suggests that ABDOM does not interfere with FtsZ polymerization *in vivo*. Thus, although the ABDOM interacts with polymerized FtsZ it does not appear to influence the degree of polymerization either *in vitro* or *in vivo*.

We then investigated the effect of the ABDOM-FtsZ interaction on the distribution of newly synthesized peptidoglycan (PG), also known as murein, in cells overexpressing MscSΔ266–286. This was done over one cell cycle by pulse labeling with a D-amino acid, followed by a chase without this amino acid and immunomicroscopy [34]. The labeled, older PG was seen as bright spots, while the more recently synthesized murein was seen as dark background areas (Fig 2, panel D). Overexpression of MscSΔ266–286 resulted in cell filamentation, with clear dark rings of newly synthesized murein in septal areas (Fig 2, panel D). A similar pattern of murein segregation was observed in cells treated with aztreonam, a selective inhibitor of PBP3 whose septal localization depends on functional Z-rings (Fig 2, panel D). These results indicate that, although, Z-ring function is disrupted and cell division is inhibited by the overexpression of MscSΔ266–286, the formation of the Z-rings and murein pre-septal rings is not affected.

The cell filamentation induced by overexpression of ABDOM or by MscSΔ266–286 is a non-physiological phenomenon; however, it represents a useful indication of the MscS-FtsZ interaction.

### Impaired MscS-FtsZ binding is associated with compromised growth on β-lactam antibiotics

**Fig 3:**
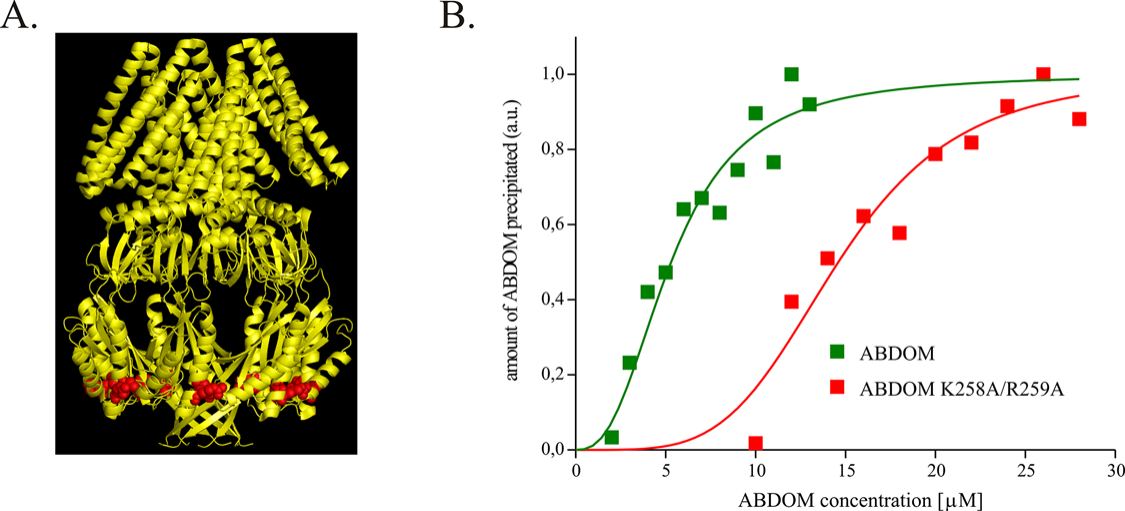
Alanine substitutions of K258 and R259 in ABDOM impair its binding to FtsZ *in vitro*. A. A crystal structure of nonconductive MscS (PDB ID: 2OAR), amino acids K258 and R259 are shown in red spacefill. **B**. Double alanine substitution K258A/R259A impaired ABDOM binding to FtsZ. Co-sedimentation experiments were carried out similarly to those shown in Fig 2, panel A. The data were fitted with Hill model (y = x^n^/(Kd+x^n^) with Kd = 5.3 μM, n = 2.6 and Kd = 14.6 μM, n = 4.3 for MBP-ABDOM and MBP-ABDOM-K258A/R259A respectively.

In order to better understand the interaction between MscS and FtsZ we searched for mutations in ABDOM that would abolish the binding. We constructed several double and triple mutants of MBP-ABDOM with alanines substituting amino acids exposed to its external surface (accordingly to the location in the crystal structure representing the nonconductive state of the channel [35]). We purified these proteins and tested their binding to FtsZ *in vitro* (S3 Fig, panel A). Among the mutations tested the double mutation K258A/R259A (S3 Fig, panel A) had the most significant effect, reducing binding affinity by approximately 3-fold (from Kd 5.3 μM in MscS to 14.6 μM in the mutant) (Fig 3, panel B and S3 Fig, panel B). Introduction of K258A/R259A to MscS did not change channel activation threshold (not shown) nor protein expression level (S1 Fig) and its ability to protect cells from osmotic downshocks (S4 Fig) indicating that the mutation did not change MscS channel properties significantly. To check whether K258A/R259A mutation impairs FtsZ-binding *in vivo* we wanted to investigate cell filamentation induced by MscSΔ266–286 overexpression, which we consider a non-physiological hallmark of the MscS-FtsZ interaction. Unfortunately, the K258A/R259A mutation introduced into MscSΔ266–286 reduced the level of protein expression dramatically (not shown) and we were unable to determine its effect on the cell filamentation *in vivo*. Since mutations K258 and R259 are situated at the very bottom of the ABDOM (Fig 3, panel A) and thus indicate a possible FtsZ binding site, we decided to introduce there a bulky tag that might sterically interfere with FtsZ binding. We fused YFP to the C-terminus of MscS and found out that MscS-YFP did not change the MscS channel properties (S4 and S5 Figs). However, we noticed that the wild-type and the YFP-tagged MscS responded in a different way to the addition of FtsZ to the cytoplasmic side of the membrane. FtsZ slowed down adaptation as well as the recovery from inactivation by 12% in the wild-type MscS and did not in the tagged MscS (S6 Fig).

Next we fused YFP to MscSΔ266–286 to investigate whether cells expressing MscSΔ(266–286)-YFP show a non-physiological filamentation. As we expected overexpression of MscSΔ(266–286)-YFP did not induce cell filamentation, unlike overexpression of the untagged MscSΔ266–286 (S7 Fig). Both lines of evidence: the one using the double K258A/R259A mutation and the other one introducing the bulky tag to the end of MscSΔ266–286 confirmed that putative ABDOM—FtsZ interaction is a prerequisite for the observed cell filamentation and show that the binding site of FtsZ is situated at the bottom of the ABDOM.

**Fig 4:**
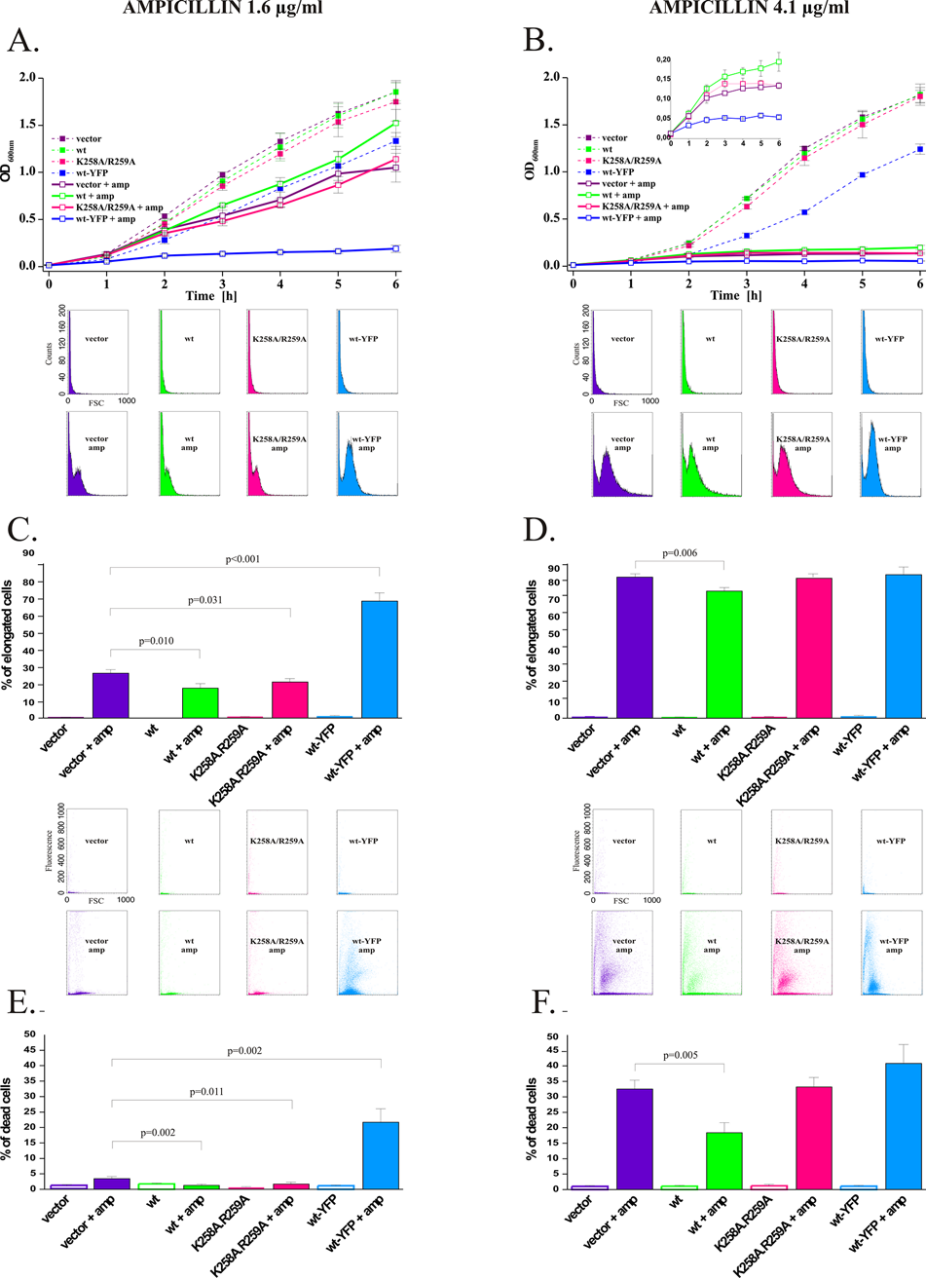
MscS protects cells from β-lactam antibiotics. Experiments were conducted on plasmid transformed MJF429 strain in two different concentrations of ampicillin: 1.6 μg/ml (**A**, **C**, **E**) and 4.1μg/ml ampicillin (**B**, **D**, **F**). **A, B.** Overexpression of MscS but not MscS-K258A/R259A or MscS-YFP supports the cell growth in the presence of subMIC of ampicillin. Inset in **B**: growth of ampicillin treated cells shown using expanded Y axis scale. **C, D.** The number of elongated cells estimated from forward scatter (FSC) was reduced for bacteria expressing MscS (green) as compared to MscS-K258A/R259A (pink) or MscS-YFP (blue). Sample FSC histograms are presented in insets, for complete FSC analysis see S8 Fig, panels A-C. **E, F.** The number of cells with compromised membrane integrity estimated using PI staining was decreased for bacteria expressing MscS (green) as compared to MscS-K258A/R259A (pink) or MscS-YFP (blue). Sample scatter plots are presented for complete FC analysis see S8 Fig, panels D-F. Cells grown in A, B were then used in experiments presented in **C**, **D**, **E**, **F**. For statistically different results p-values < 0.05 of paired Student`s t-test are shown above the graphs (n = 3).

FtsZ is known to be responsible for cell division but also directs cell wall synthesis [36]. We wondered, whether MscS could be responsible for protection against cell wall damage and whether this function requires interactions with FtsZ. Ampicilin is a broad spectrum ß-lactam penicillin that binds to all penicillin binding proteins [37] and by inhibiting PG cross-linking induces general lesions of peptidoglycan [38]. Penicillin treatment often results in membrane protrusions emanating from midcell before they rupture [39, 40]. An accepted hypothesis to explain this is that penicillin causes a defect in the splitting of septal PG during division. Consistent with this idea, blocking of cell division has been shown to have protective effects when *E*. *coli* cells are treated with ß–lactams [41]. FtsZ is a central division protein that directs the septal as well as the lateral wall PG synthesis [34, 36]. We expected that if indeed MscS functionally interacts with FtsZ, it could also have protective effect via this interaction against ampicillin treatment. To induce cell wall lesions without cell lysis we used of ampicillin in concentrations below minimal inhibitory concentration (subMIC) in which we did not observe any apparent cell morphology changes. We transformed mscS^-^ strain MJF429 with pWG1 plasmids (kanR derivatives of pTRC99A) carrying *mscS* alleles. We found that overexpression of MscS, but not MscS-K258A/R259A nor MscS-YFP, increases the growth rate of cells cultivated in the presence of subMIC of ampicillin (lower—1.6 μg/ml or higher—4.2 μg/ml) (Fig 4, panels A and B). These results suggest that MscS protects cells against ampicillin and the lack of this effect in MscS-K258A/R259A or in MscS-YFP suggests that the mechanism of protection is associated with the MscS binding of FtsZ. To get more insight into this mechanism we investigated the ampicillin treated cells by flow cytometry (FC). We measured forward scatter (FSC) which is proportional to the cell length [42]. We noticed that ampicillin treatment resulted in a new population of cells with increased length in all strains tested (Fig 4, panels C and D, see insets above the plots). Fractions of elongated cells in the presence or in the absence of ampicillin are shown as bars on X axes in Fig 4, panels C and D. Larger number of the elongated cells was observed in higher concentration of ampicillin (Fig 4, panel D). It was demonstrated previously that subMIC of ampicillin arrested *E*. *coli* cell division by inducing SOS system [41] what in turn resulted in cell elongation [43]. Curiously, in our experiments expression of wt-MscS partially relieved this effect (Fig 4, panels C andD; S8 Fig, panels A-C). In cells expressing MscS, both the percent of elongated cells and the median cell scatter (S2 Table) were lower compared to those in control, while the protective effect of expression of MscS-K258A/R259A was weaker (Fig 4, panels C and D; S8 Fig, panels A-C). In the presence of 1.6 μg/ml ampicillin expression of MscS-YFP resulted in the largest number of elongated cells (Fig 4, panel C) as a consequence of almost complete cell division arrest (Fig 4, panel A). Similar phenomenon was seen for all constructs in 4.2 μg/ml ampicillin (cell elongation is shown in Fig 4, panel D and cell division arrest is shown in Fig 4, panel B).

Growth in ampicillin results in PG damage. Therefore we wondered whether we could see decreased cell envelope integrity under our experimental conditions. To this end we stained cells with propidium iodide (PI) [44] which is excluded from live cells but stains the damaged and dead cells (Fig 4, panels E andF see insets above the plots, and S8 Fig, panels D-F). In the higher concentration of ampicillin (Fig 4, panel F) the fractions of stained cells (displayed as bars on X axes) were larger than those in the lower ampicillin concentration (Fig 4, panel E). In the higher ampicillin concentration expression of MscS resulted in fewer dead or damaged cells (18.42% ± 3.24% vs. 32.58% ± 2.80% in control), while expression of MscS-K258A/R259A mutant did not have any effect (33.23% ± 3.06%) (Fig 4, panel F). It is worth to note that expression of MscS-YFP sensitized cells against ampicillin treatment (40.89% ± 6.01% of PI stained cells). This effect was even more pronounced in the lower ampicillin concentration: expression of MscS-YFP resulted in 21.76% ± 4.27% of PI stained cells as opposed to 3.52% ± 0.42% and 1.35% ± 0.33% of PI stained cells carrying vector and expressing MscS, respectively.

## Discussion

We have provided physiological and biochemical evidence that ABDOM—a domain in the cytoplasmic C-terminal part of the MscS channel—binds FtsZ, the bacterial homolog of tubulin, a protein crucial to cell division. We have also demonstrated that the MscS—FtsZ interaction that occurs upon non-conducting inactivated state of that channel is involved in cell protection against antibiotic stress.

MscS has been shown to protect against severe osmotic down-shocks [45]. It was also suggested that MscS helps cells to accommodate to the rise in membrane tension that occurs both during the stationary phase and upon exit from the stationary phase as a result of cell wall remodeling [46]. Until now, based on experimental evidence, it was thought that these functions depended entirely on the ability of MscS to conduct ions in order to relieve osmotic stress. However, while this makes sense for protecting cells against brief and strong osmotic shocks, it poses a problem during sustained stress and over prolonged periods of cell wall remodeling. Indeed, to prevent the depletion of the ion gradient, sustained periods of membrane tension lead to inactivated states of MscS [47]. After opening and release of solutes upon osmotic down-shock, MscS undergoes a slow transition (seconds) into a non-conducting inactivated state [47] and therefore, one would expect a low occupancy of the inactivated state after sudden osmotic down-shock (when cell turgor pressure falls rapidly and the MscS gates close). MscS also enters the inactivated state (C→I transition) directly from the closed state [8]. This can be seen easily in patch-clamp experiments when suction is applied slowly to the recording pipette [6], or the pressure is held just below the activation threshold (S9 Fig, panel A). The C→I transition has an apparent lower threshold than that for the opening (the tension thresholds for opening and inactivation are similar, but the opening rate has a steeper tension dependence [6,8]) (S9 Fig, panel B) and it is therefore more probable under stress conditions that produce moderate or slowly rising membrane tension. Moreover, as found in electrophysiological experiments, recovery from inactivation of MscS requires a release of tension [8] and consequently sustained subthreshold stimulus keeps channels in an inactivated state (S9 Fig, panel A). Such stimuli could arise during growth or entry into the stationary phase or when the cell envelope bulges as a result of PG damage (see further in Discussion).

It has been demonstrated in patch-clamp experiments that the MscSΔ266–286 mutation (removing the β-barrel at the bottom of the channel) results in a unique behavior: after opening the channel inactivates but could not be activated ever again [15]. Previous work suggests that MscS gating is coupled to conformational changes of its cytoplasmic chamber [14,18–20] and these are not possible without some movement of the β-barrel [14]. We think that removal of the β-barrel in MscSΔ266–286 could result in the channel that is trapped in an inactivated-like state during normal cell growth. This assumption is supported by the observation that the protection of MscSΔ266–286 against osmotic downshocks is partial [15]. Alternatively, opening of MscSΔ266–286 could result in a tension-induced misfolded state with non-native quaternary structure. Irrespectively of the exact conformation of the MscSΔ266–286 it seems unlikely that removal of β-barrel alone could result in misfolding of a distinct domain—ABDOM, since all the amino acids at contact sites between ABDOM`s are preserved [48]. Similarly, native ABDOM conformation seems to be retained in the isolated ABDOM, since similar cellular effect (filamentation) is observed for both MscSΔ266–286 and isolated ABDOM. Therefore, it is likely that binding of FtsZ to MscS depends on the exposure of a binding site that is hidden in the closed state of the channel. We carried out patch-clamp experiments in which we applied FtsZ to the cytoplasmic side of membrane patches. This slowed down MscS channel adaptation as well as recovery from inactivation. This effect was not observed when MBP, a protein of similar mass and charge was applied. Interestingly FtsZ did not have impact on MscS-YFP activity, indirectly indicating for lack of interaction with FtsZ. These experiments indicate that both open and inactivated state of the channel are affected by FtsZ and indirectly indicate that in both states MscS is proficient at binding of FtsZ. However, as argued above MscS stays open only for brief time periods *in vivo*, while inactivated state could be more prevalent. It is therefore the inactivated state in which FtsZ binding could have physiological impact.

What is the mechanism by which overexpression of MscSΔ266–286 mutant interferes with cell division? We assume that widespread binding of FtsZ by MscSΔ266–286 sponges up FtsZ and causes non-physiological cell filamentation while the wt-MscS upon its normal inactivation would anchor FtsZ only locally at sites of local membrane stress. Isolated ABDOM does not block FtsZ polymerization *in vitro* and MscSΔ266–286 does not prevent formation of Z-rings *in vivo* indicating that cell elongation induced by MscSΔ266–286 is rather a result of its interference with further steps of assembly of division machinery.

We showed, by introducing K258A/R259A mutation slightly impairing the ABDOM—FtsZ interaction that FtsZ binds close to the very bottom of the MscS chamber, just near the β-barrel. Overexpression of MscS-YFP had a dramatic effect leading to growth arrest in the media with ampicillin. We believe that this phenotype is due to disruption of the ABDOM-FtsZ interaction by YFP, which creates a steric obstacle. Even though we identified specific interaction of FtsZ with isolated ABDOM, this is not a proper model of the interaction between these two proteins, which probably depends on the quaternary structure of MscS. Therefore, any binding studies between FtsZ and isolated ABDOM fused to YFP are not relevant. On the other hand we were not able to detect interaction of MscSΔ266–286 and FtsZ with membrane two hybrid system [49] or by bimolecular fluorescence complementation [50]. Further studies to directly asses interaction of FtsZ with ABDOM in the context of the channel (including MscS-YFP) are therefore needed. A method of choice could be a use of nanodiscs to incorporate MscS into soluble membrane environment and to study association of such nanodisc/channel complexes with polymerized FtsZ [51,52]. We think that the negative effect of overexpression of MscS-YFP in the presence of subMIC ampicillin indicates that another system of cell defense against ampicillin, other than that provided by MscS, was displaced by the excess of MscS-YFP from the sites of damage. Our FACS analysis proved that in the presence of ampicillin overexpression of wt-MscS but not MscS-K258A/R259A or MscS-YFP reduces the number of elongated and damaged (PI permeable) cells. Other authors found out that treatment of *E*. *coli* with subMIC ampicillin induced SOS system via PBP3 inhibition and this led to a block of cell divisions resulting in cell filamentation [41]. We observed lower number of elongated cells, when MscS was expressed and it could indirectly indicate to the lower level of SOS induction probably as a result of lower level of PG damage.

**Fig 5:**
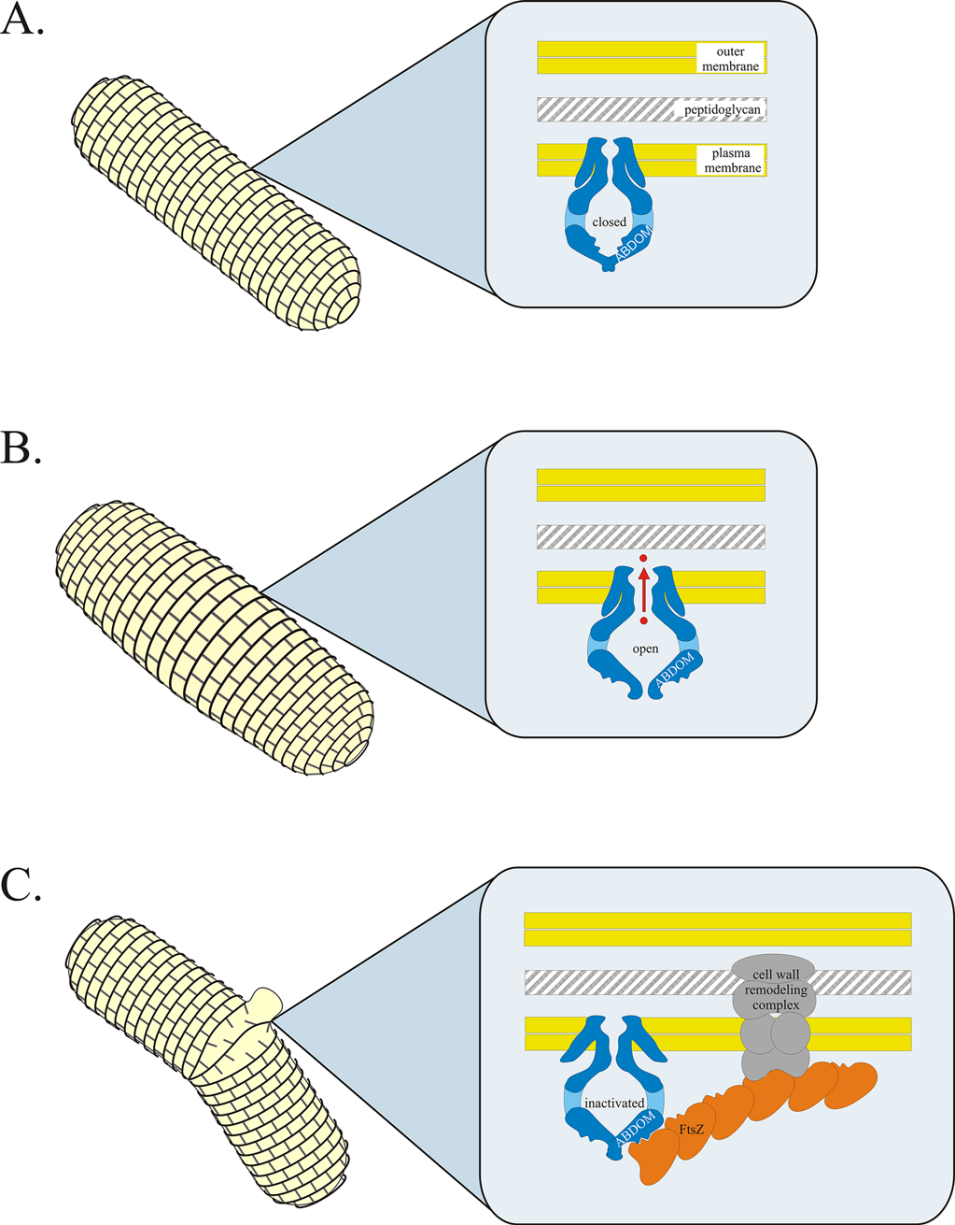
Hypothetical model of MscS acting as a dual-action protective gate. A. Under non-stress conditions MscS remains in the closed state and the ABDOM domain of MscS is inaccessible to interaction with FtsZ. **B.** Under severe osmotic downshocks the channel opens and jettisons osmolytes rapidly returning to the closed state. The short-lived open state does not result in formation of the cell-wall remodeling complex. **C.** Under slowly rising and prolonged local increase of tension within the bulges of the membrane the channel performs transition into the inactivated state. In this channel conformation ABDOM becomes accessible and binds FtsZ.

We have demonstrated that MscS protects cells against antibiotic stress and this function is independent of its ability to protect against osmotic downshocks. We believe that MscS acts here as an interpreter of local changes of membrane tension due to small lesions induced by β-lactam antibiotics. The question arises as to how MscS channel can sense local envelope stress. It is well established that Gram-negative PG is a single layer mesh formed by long strands of glycans cross-linked by stretchable peptides [53–56]. This forms a structure with pores known as “tesserae” [55]. The existing models predict that breaking peptide cross-links leads to defects which eventually end up with cell lysis. However, before it occurs the turgor pressure expands the envelope around the defects to form “bulges”. Such bulges or blebs have been found in in the midcell of cells treated with β-lactam antibiotics [37,57,58] but also cells undergoing slow osmotic downshocks [59] indicating for susceptibility of the cell wall at the division site. It is very likely that the membrane of the bulges contains MscS protein. In fact it was demonstrated that MscS prefers to localize in an osmotic-stress—dependent manner at cell poles in cardiolipin (CL) [60]. CL shows preference to curved membranes and it was shown that CL forms micro-domains in *E*. *coli* membranes in response to curvature [61]. It is therefore reasonable to assume that bulges will contain CL micro-domains which in turn could result in preferential localization of MscS in the bulges. It is well established that MscS gates in response to tension and curvature [11], which are different in the bulge than in the rest of the cell, and the specific localization of MscS allows the channel to sense tension only locally, and possibly, to direct FtsZ for local PG repair. Other data also indicate that tension affects PG synthesis: i. the curved rod shape of *Caulobacter crescentus* depends on the presence of intracellular filament of crescentin attached to the membrane [62], ii. crescentin expressed in *E*. *coli* makes the cells curved as a result of crescentin-dependent gradient of PG insertion around the cell circumference [63]. In our model we propose that MscS plays active role by recruiting FtsZ to the sites of damage, what in turn can direct cell wall synthesi/repair (Fig 5). However, we cannot exclude entirely the possibility that the role of MscS is passive and local binding of FtsZ blocks division as long as the cell wall is not repaired by an independent mechanism. This could be supported by observations that blocking of cell division has protective effects when *E*. *coli* cells are treated with some β-lactams [39,64,65]. Despite the fact that we do not know exact mechanism by which MscS cooperates with FtsZ to fulfill cellular function, we believe that MscS-FtsZ interaction is important in specific stress conditions. We have to note that we were not able to prove that native expression of MscS protects against β-lactams (not shown). However, the effect could be subtle and masked by other mechanisms. Similarly, there are multiple MscS paralogs of MscS in *E*. *coli* and initial experiments suggested that they cannot protect cells against osmotic downshocks unless overexpressed [66]. However, recently it was found that the protection provided by MscS homologs depends strongly on the rate of osmotic downshock and under a slow enough osmotic drop, MscS homologs can lead to survival rates comparable to those found in wild-type strains [67].

There are multiple studies to date on the role of mechanosensitive channels in osmotic regulation. These include protection of *E*. *coli* against osmotic downshocks by MscL and MscS and its paralogs [67], as well as protection of *Arabidopsis thaliana* plastids against hypoosmotic stress by MSL2 and MSL3, the MscS-like proteins [68]. These functions were ascribed solely to the ability of these proteins to open under tension and to release solutes. There are, however, other experimental indications that some MscS—like proteins may not be involved in processes solely attributed to protection against osmotic imbalance. For example, it was found that PamA, an MscS-like protein in *Synechocystis sp*., binds cytoplasmic protein PII responsible for coordination of signal transduction pathways associated with nitrogen and carbon status, and *pamA* mutants are unable to grow in glucose containing media [69]. In *A*. *thaliana* overexpression of MscS paralog—MSL10 is responsible for induction of cell death and it was identified that expression of only N-terminal domain of the channel was enough to exert this effect [70]. Our results also contribute to these studies. We demonstrate here that ABDOM, a domain of MscS cytoplasmic part, binds FtsZ, the central division protein and the mutations affecting the binding influence cell shape and/or division. Studies of the same kind done on *A*. *thaliana* revealed that MSL2 and MSL3 are implicated in shape regulation and division of chloroplasts of *A*. *thaliana* [71]. MSL2 and MSL3 colocalize with plastid division protein AtMinE and msl2 msl3 double mutants have enlarged chloroplasts containing multiple FtsZ rings (as opposed to a single FtsZ ring in wild-type chloroplast). Genetic analyses pointed out that MSL2, MSL3, and components of the Min system function in the same pathway to regulate chloroplast size and FtsZ ring formation [72]. Our data strongly suggest that similar connection is present in *E*. *coli*. This statement is also supported by the recently described observation that *E*. *coli* strain lacking both MscS and MscL channels, exhibited aberrant FtsZ ring assembly [73].

In summary: based on our results we hypothesize that MscS functions as a dual-action protective gate for coping with osmotic stress (schematic representation in Fig 5), with the already understood function that channel opens and conducts solutes providing rapid, short-lived, accommodation to osmotic stress, and with a second, novel mechanism described here in which channel inactivation converts MscS into a membrane anchoring protein that triggers cell wall repair/remodeling to protect against sustained stress.

## Supporting Information

S1 Fig Expression of the wild-type MscS and its mutants. MJF429 cells expressed MscS variants tagged at the C-termini with HA epitope. **A.** The HA tagged proteins were detected with monoclonal HA antibody. Note the low intensity of the band corresponding to ABDOM-HA. **B.** Corresponding band was clearly visible on Coomassie Blue stained gel.
(TIF)

S2 Fig Purity of recombinant proteins. SDS-PAGE of FtsZ, MBP-ABDOM and MBP. 1 or 3 μg of each protein loaded per lane. Gel stained with Coomassie Brilliant Blue (Merck).
(TIF)

S3 Fig Analysis of the binding of the wild-type and the mutants of MBP-ABDOM to GTP-polymerized FtsZ. FtsZ was precipitated in the presence of the indicated proteins as described in the Experimental Procedures. The amount of co-precipitated proteins was assessed by Western blotting with anti-MBP antibody. **A.** Analysis of co-precipitation of the wild-type and the mutants of MBP-ABDOM in the absence (-) or in the presence (+) of 16 μM FtsZ. All samples contained 2 mM GTP and 8 μM MBP or MBP-ABDOM variant as indicated. **B.** Analysis of co-precipitation of increasing concentration of MBP-ABDOM (4, 8 and 12 μM) and its mutants K258A or K258A/R259A with 16μM FtsZ. In the first two lanes MBP-ABDOM (8 μM) was incubated with (+) or without (-) GTP. **C.** Analysis of co-precipitation of 8 μM MBP, MBP-ABDOM or MBP-ABDOM-R259A with increasing concentration of FtsZ (0, 8 and 16 and 32 μM).
(TIF)

S4 Fig MscSΔ266–286, MscS-K258A/R259A and MscS-YFP retain ability to protect cells from osmotic downshocks. MJF465 cells transformed with pTRC99A or its derivatives carrying *mscS* variants were grown without inducer and tested according to standard protocol.
(TIF)

S5 Fig MscS-YFP channel behaves as wild-type MscS. Channel activity recorded from cells expressing MscS (left column) or MscS-YFP (right column). Upper panel: three different constant pressure pulses were applied to the patch and multiple channel responses were recorded. The inactivation rates of wt-MscS and MscS-YFP are similar. Lower panel: one variable pressure pulse was applied to the patch. The activation thresholds (arrows) of wt-MscS and MscS-YFP are the same.
(TIF)

S6 Fig FtsZ affects kinetics of the wild-type MscS but not the MscS-YFP. A. FtsZ slows down the rate of adaptation of the wild-type MscS (middle panel of the upper row). This effect was also not observed when MBP, protein of similar mass and charge was applied (middle panel of the lower row). Representative experiment out of four for each FtsZ and MBP is shown. **B.** FtsZ slows down the rate of recovery from inactivation of the wild-type MscS but does not change it in the MscS-YFP. Diagram on the right shows change (in percent) of the rate of recovery from inactivation in MscS in the presence of FtsZ or MBP, and in MscS-YFP in the presence of FtsZ. P-values are smaller than 0.05 (n = 4). A representative experiment showing recovery from inactivation of MscS in control (black) and after application of FtsZ (red) is shown on the left. **C.** FtsZ does not slow down the rate of adaptation of the MscS-YFP (middle panel). Representative experiment out of four is shown.
(TIF)

S7 Fig FtsZ binding depends on the conformation of MscS channel and its mutants. **C-terminal YFP is a steric obstacle for the FtsZ binding to MscSΔ266–286. A.** Cartoons of conformational changes of MscS, MscSΔ266–286 and MscS-YFP during their closed–to–inactivated transitions (cartoons were drawn according to [17]), and resultant FtsZ binding. Arrows indicate possible kinetic transitions of the channel. In each case the direction of the thick arrow indicates the more probable channel conformation. Under non-stress conditions MscS (upper row) resides in closed state that is noncompetent in FtsZ binding. Under the same conditions MscSΔ266–286 (middle row) resides in a permanent inactivated state, which makes the FtsZ binding possible. We assume that the binding of FtsZ is chronic and it results in cell filamentation. Fusing YFP to C-terminus of MscSΔ266–286 (lower row) prevents FtsZ binding and prevents cell filamentation. **B.** Microscopic images of cells expressing corresponding constructs (bright field on the left, fluorescence on the right).
(TIF)

S8 Fig Flow cytometry analysis indicates that MscS, but not MscS-K258A/R259A or MscS-YFP protects cells in the presence of ampicillin. In **A**, **B**, **C** forward scatter histograms are displayed. M1 and M2 ranges refer to two populations of cells short and long ones, respectively. Under control conditions (**A.**) only short cells were observed (M1 range). In the presence of ampicillin (**B**., **C.**) longer cells were observed additionally (M2 range). Lowest number of longer cells was observed in wt MscS. The borderline between M1 and M2 range was set manually to assign >90% of control cells to M1. In **D**, **E**, **F** fluorescence scatter plots are displayed. Damaged cells were stained with propidium iodide (PI). Cells with fluorescence above the background were counted as damaged cells (R1 area). The lowest number of damaged cells was observed in wt MscS. The level of fluorescence of unstained cells (horizontal line in each sample) was manually set as a fluorescence background (as seen in samples presented in **E**, **F** rightmost column) and was kept constant for all samples. Cells were grown in LB alone (**A**, **D**), and in LB with low (1.6 μg/ml; **B**, **E**) or high (4.1 μg/ml; **C**, **F**) concentration of ampicillin. All samples presented in **A**, **B**, **C**, **D**, **E**,
(PDF)

S9 Fig In the wild-type MscS the closed-to-inactivated transition is possible at subthreshold pressures. **A.** Experimental protocol: 1) Primary test: application of saturating pressure (p_sat_) results in I_max1_, which is a total current of all open MscS channels present in the patch. 2) Main test: after 2 min MscS traces were recorded with various preconditioning 30s-pressure pulses (arrow marks their application). At subthreshold pressures p_i =_ sub, MscS channels do not open, but some of them inactivate and do not open when saturating pressure is applied (I_max2_<I_max1_). 3) Final test, identical to the primary test: after 2 min at zero pressure the channels recover from the inactivated state and they reopen (I_max3_ = I_max1_). Then we calculated a probability of inactivation P_i_ = 1- I_max2_/I_max1,_ which is plotted in (**B.**) (white squares). Electrophysiological procedures were similar to those described previously. **B.** The probability of inactivation (P_i_), open probability (P_o_), and current (I) normalized to maximal current (I_max_) are shown as functions of pressure normalized to the activation threshold (95 mmHg in this patch). Note that P_i_ is higher than P_o_ at low applied pressures. The mean single-channel open probability (*P*_o_) during the pressure pulse was calculated by integrating the current passing through all active channels (*I)* during the pulse and dividing this integral by the current through a single open channel (*i)* and number of active channels (*N)* according to the formula *P*_o_ *= I/Ni*.
(TIF)

S1 Table Identification of proteins interacting with ABDOM by mass spectrometry. Peptides identified only in the samples from MBP-ABDOM but not from MBP pull-down are shown. The table lists peptides with scores >75 from both experiments described in Materials and Methods. We selected FtsZ for further experimentation since: i) ClpX interacts with FtsZ suggesting that its binding to ABDOM may be indirect (i.e. mediated through FtsZ), and, moreover, we observed the ABDOM-induced filamentation in *clpX* ^*-*^ strain (data not shown); ii) we observed the ABDOM-induced filamentation in *recA* ^*-*^ strain (data not shown); iii) MreB was identified with a low score and interference with its function results in round cells and not in the cell filamentation. Moreover, MreB possibly associates with FtsZ, so that its interaction with ABDOM could be indirect.
(DOCX)

S2 Table Percent of elongated cells and median cell scatter of *E*. *coli* cells treated with ampicillin. Experimental data from S8 Fig, panel C were analyzed and percent of elongated cells (M2 range) and median cell scatter is shown for cell treated with 4.1 μg/ml ampicillin.
(DOCX)
