# Supplementary figures and images for: Cytoplasmic Domain of MscS Interacts with Cell Division Protein FtsZ: A Possible Non-Channel Function of the Mechanosensitive Channel in Escherichia Coli
Source: PLoS One. 2015 May 21;10(5):e0127029. doi: 10.1371/journal.pone.0127029 (PMC4440785; doi:10.1371/journal.pone.0127029)

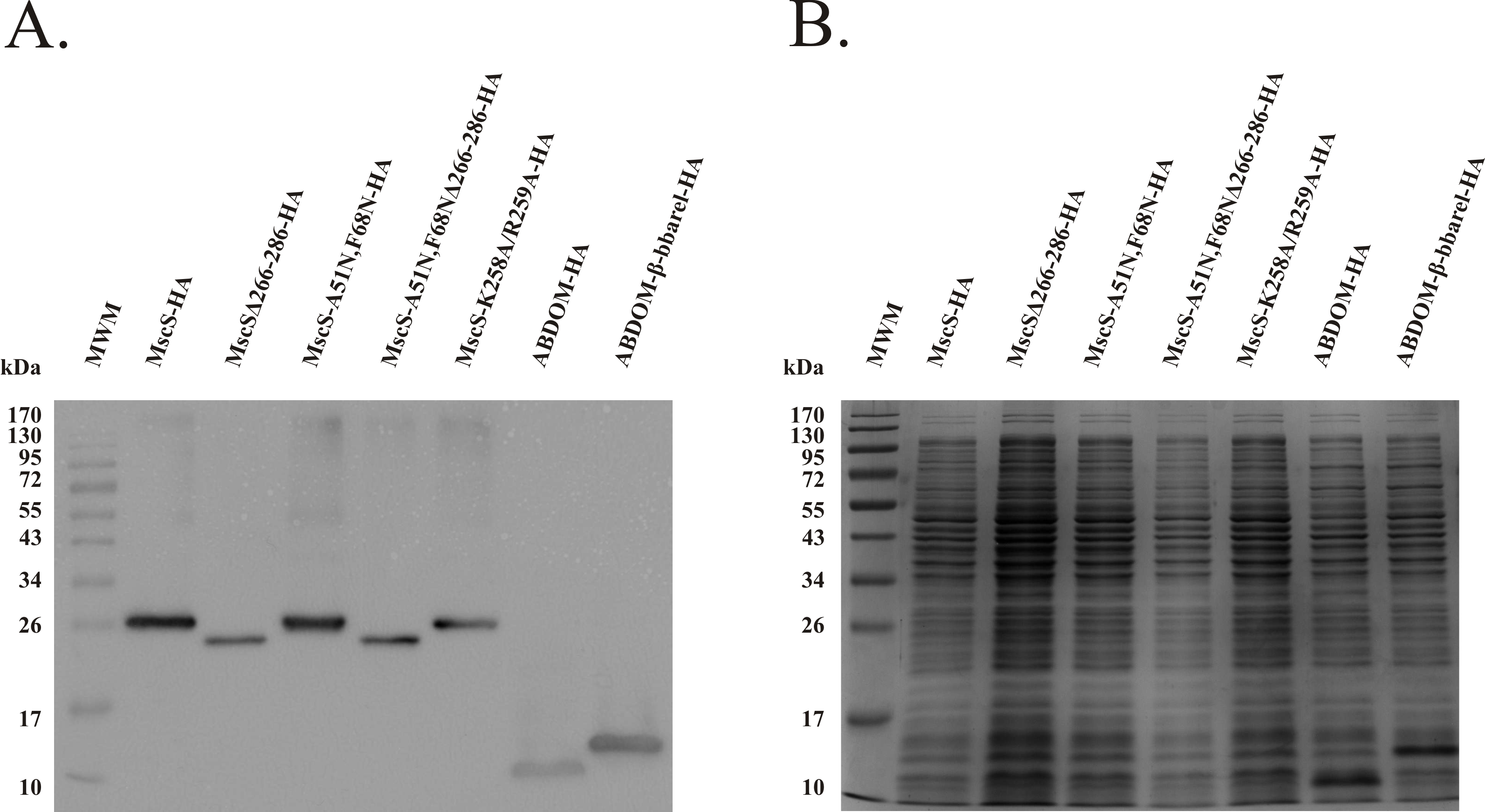

Supplement: S1 Fig — MJF429 cells expressed MscS variants tagged at the C-termini with HA epitope. A. The HA tagged proteins were detected with monoclonal HA antibody. Note the low intensity of the band corresponding to ABDOM-HA. B. Corresponding band was clearly visible on Coomassie Blue stained gel. (TIF) [file pone.0127029.s001.tif]

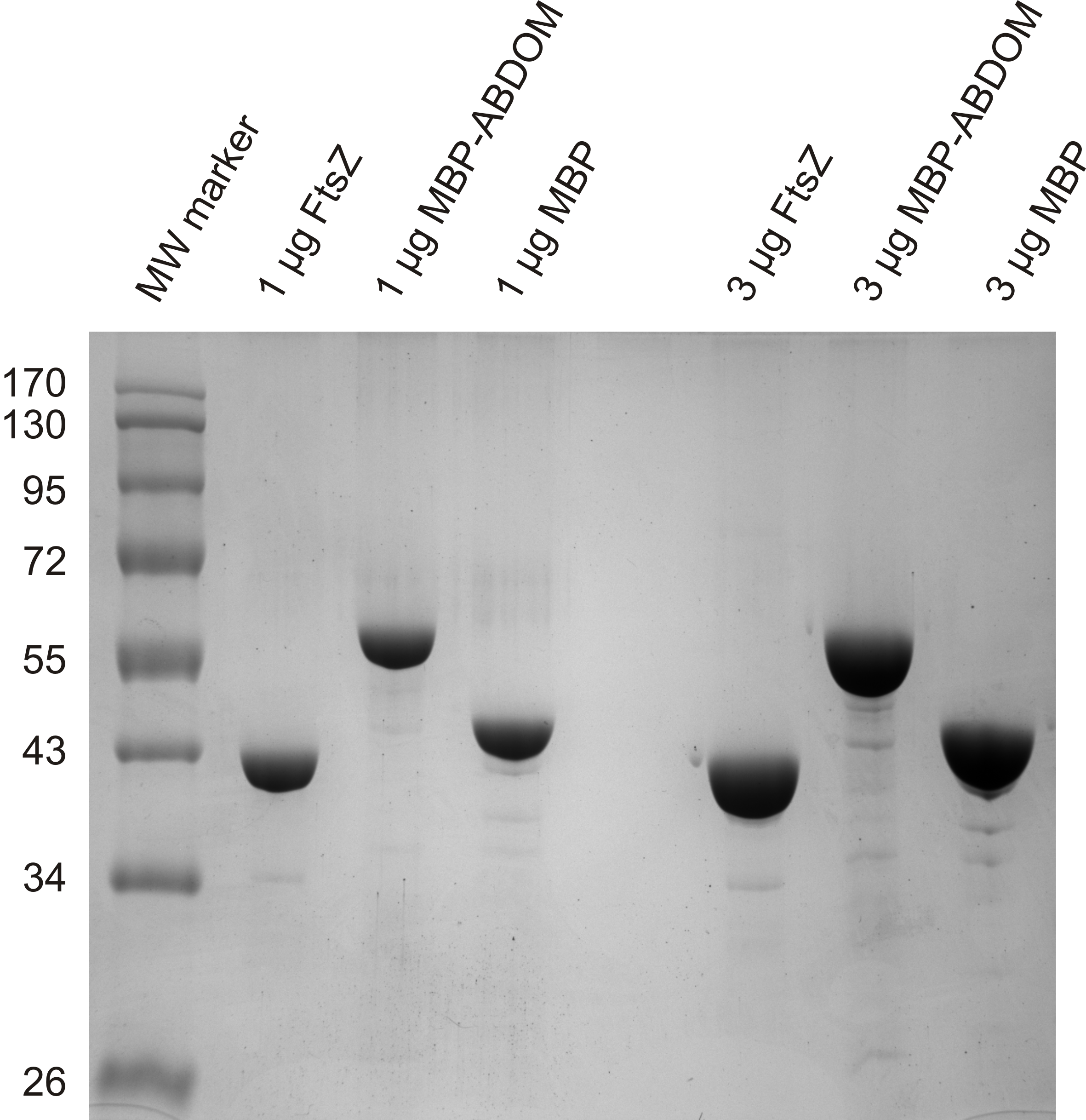

Supplement: S2 Fig — SDS-PAGE of FtsZ, MBP-ABDOM and MBP. 1 or 3 μg of each protein loaded per lane. Gel stained with Coomassie Brilliant Blue (Merck). (TIF) [file pone.0127029.s002.tif]

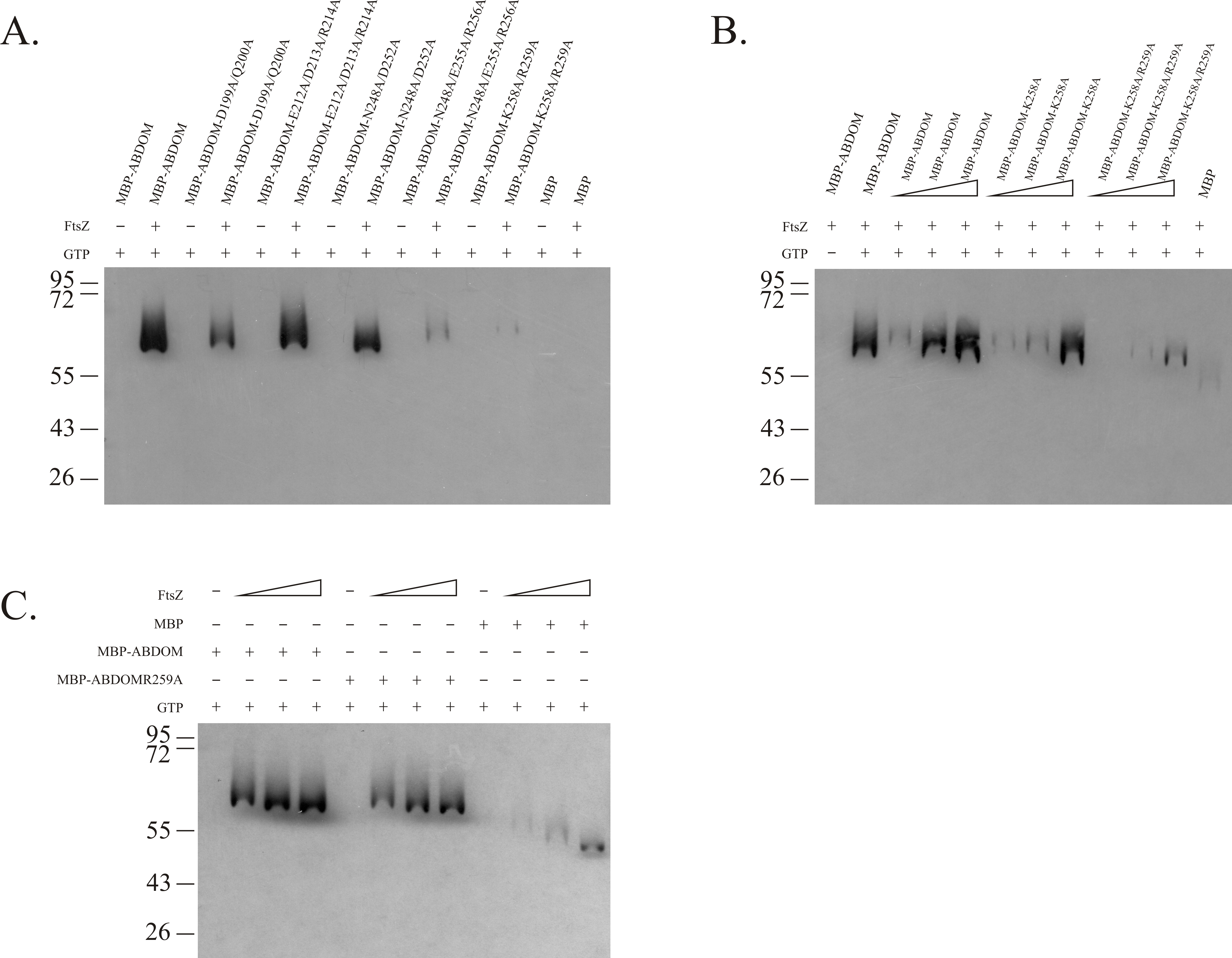

Supplement: S3 Fig — FtsZ was precipitated in the presence of the indicated proteins as described in the Experimental Procedures. The amount of co-precipitated proteins was assessed by Western blotting with anti-MBP antibody. A. Analysis of co-precipitation of the wild-type and the mutants of MBP-ABDOM in the absence (-) or in the presence (+) of 16 μM FtsZ. All samples contained 2 mM GTP and 8 μM MBP or MBP-ABDOM variant as indicated. B. Analysis of co-precipitation of increasing concentration of MBP-ABDOM (4, 8 and 12 μM) and its mutants K258A or K258A/R259A with 16μM FtsZ. In the first two lanes MBP-ABDOM (8 μM) was incubated with (+) or without (-) GTP. C. Analysis of co-precipitation of 8 μM MBP, MBP-ABDOM or MBP-ABDOM-R259A with increasing concentration of FtsZ (0, 8 and 16 and 32 μM). (TIF) [file pone.0127029.s003.tif]

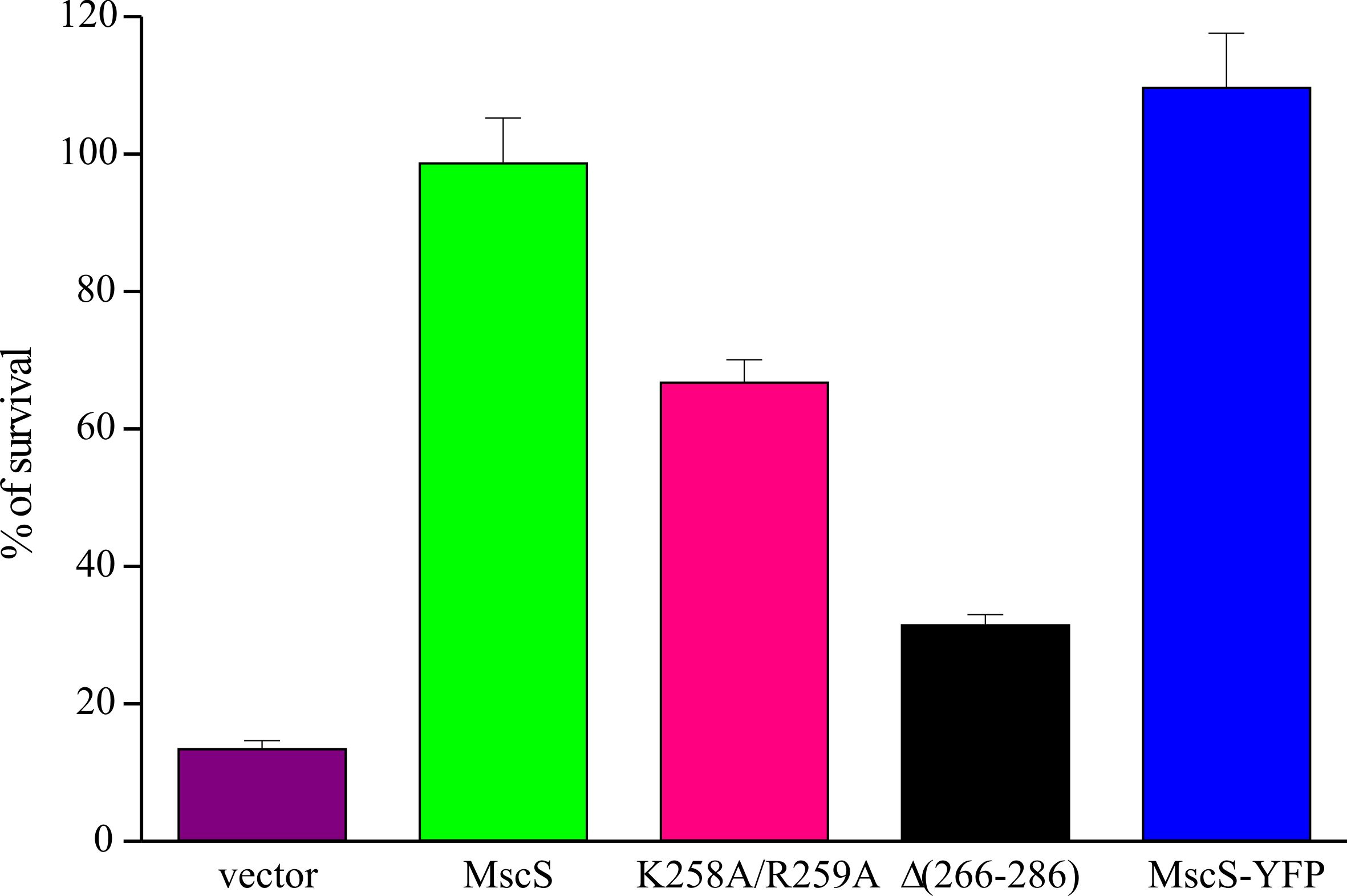

Supplement: S4 Fig — MJF465 cells transformed with pTRC99A or its derivatives carrying mscS variants were grown without inducer and tested according to standard protocol. (TIF) [file pone.0127029.s004.tif]

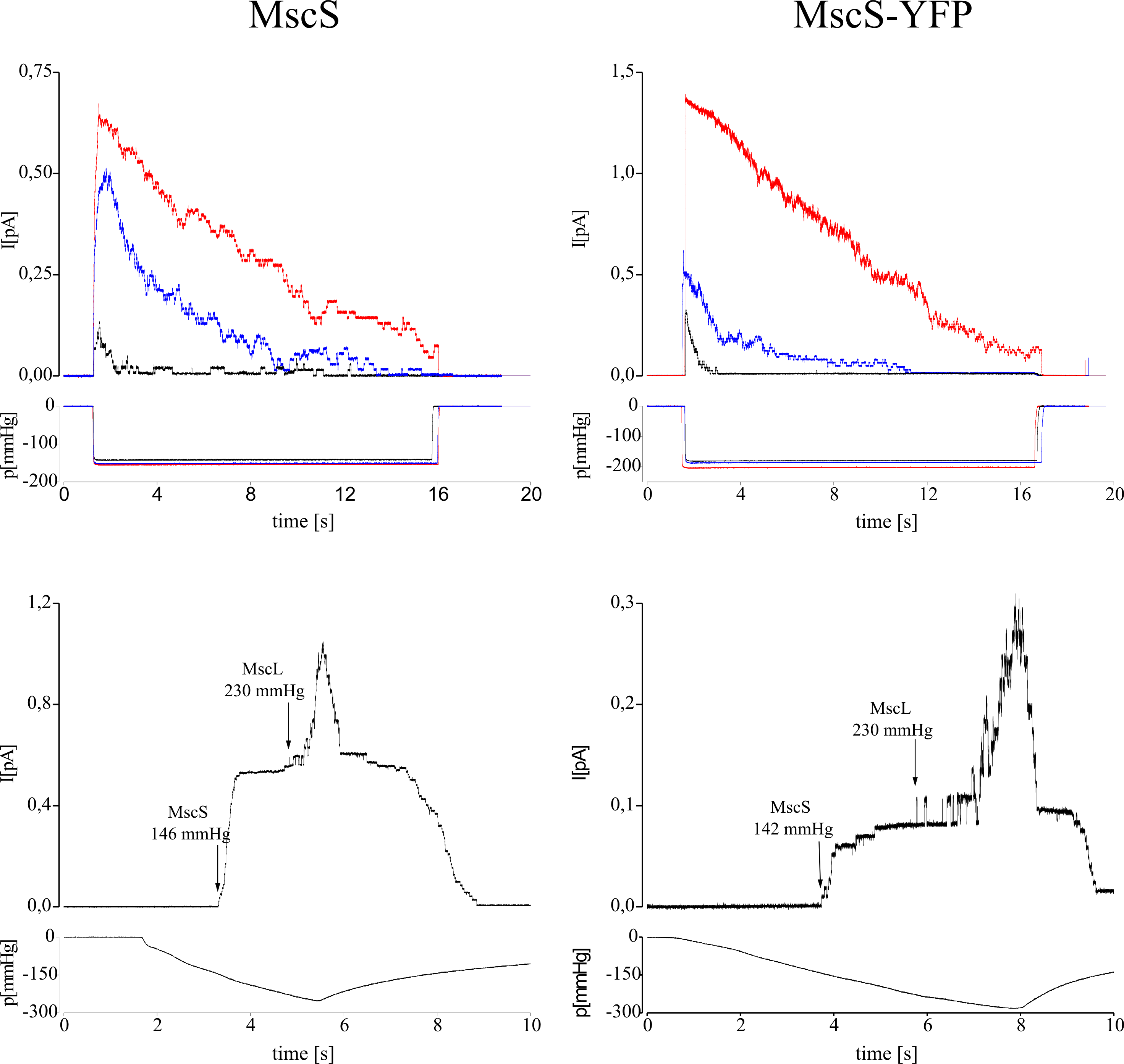

Supplement: S5 Fig — Channel activity recorded from cells expressing MscS (left column) or MscS-YFP (right column). Upper panel: three different constant pressure pulses were applied to the patch and multiple channel responses were recorded. The inactivation rates of wt-MscS and MscS-YFP are similar. Lower panel: one variable pressure pulse was applied to the patch. The activation thresholds (arrows) of wt-MscS and MscS-YFP are the same. (TIF) [file pone.0127029.s005.tif]

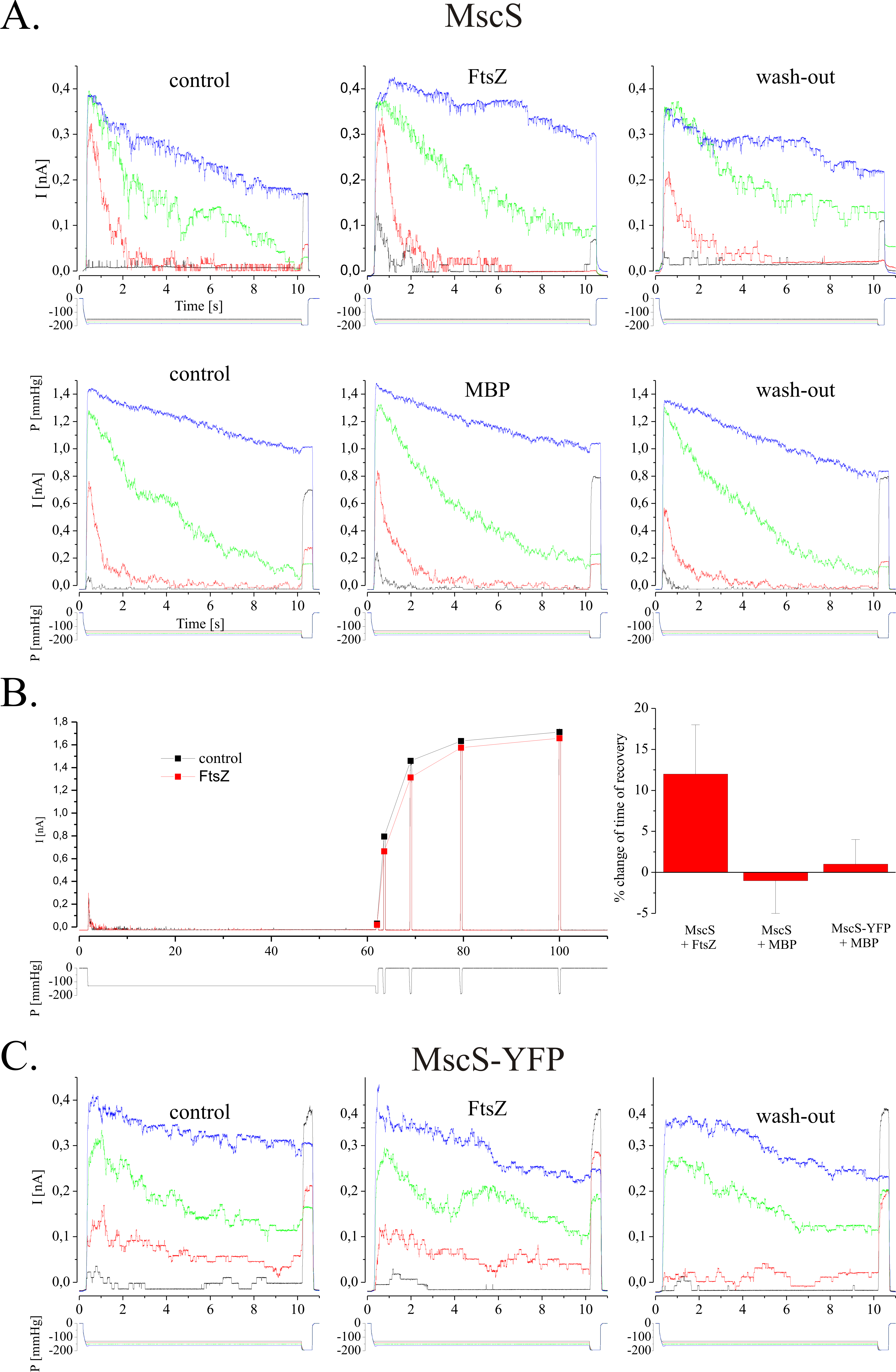

Supplement: S6 Fig — A. FtsZ slows down the rate of adaptation of the wild-type MscS (middle panel of the upper row). This effect was also not observed when MBP, protein of similar mass and charge was applied (middle panel of the lower row). Representative experiment out of four for each FtsZ and MBP is shown. B. FtsZ slows down the rate of recovery from inactivation of the wild-type MscS but does not change it in the MscS-YFP. Diagram on the right shows change (in percent) of the rate of recovery from inactivation in MscS in the presence of FtsZ or MBP, and in MscS-YFP in the presence of FtsZ. P-values are smaller than 0.05 (n = 4). A representative experiment showing recovery from inactivation of MscS in control (black) and after application of FtsZ (red) is shown on the left. C. FtsZ does not slow down the rate of adaptation of the MscS-YFP (middle panel). Representative experiment out of four is shown. (TIF) [file pone.0127029.s006.tif]

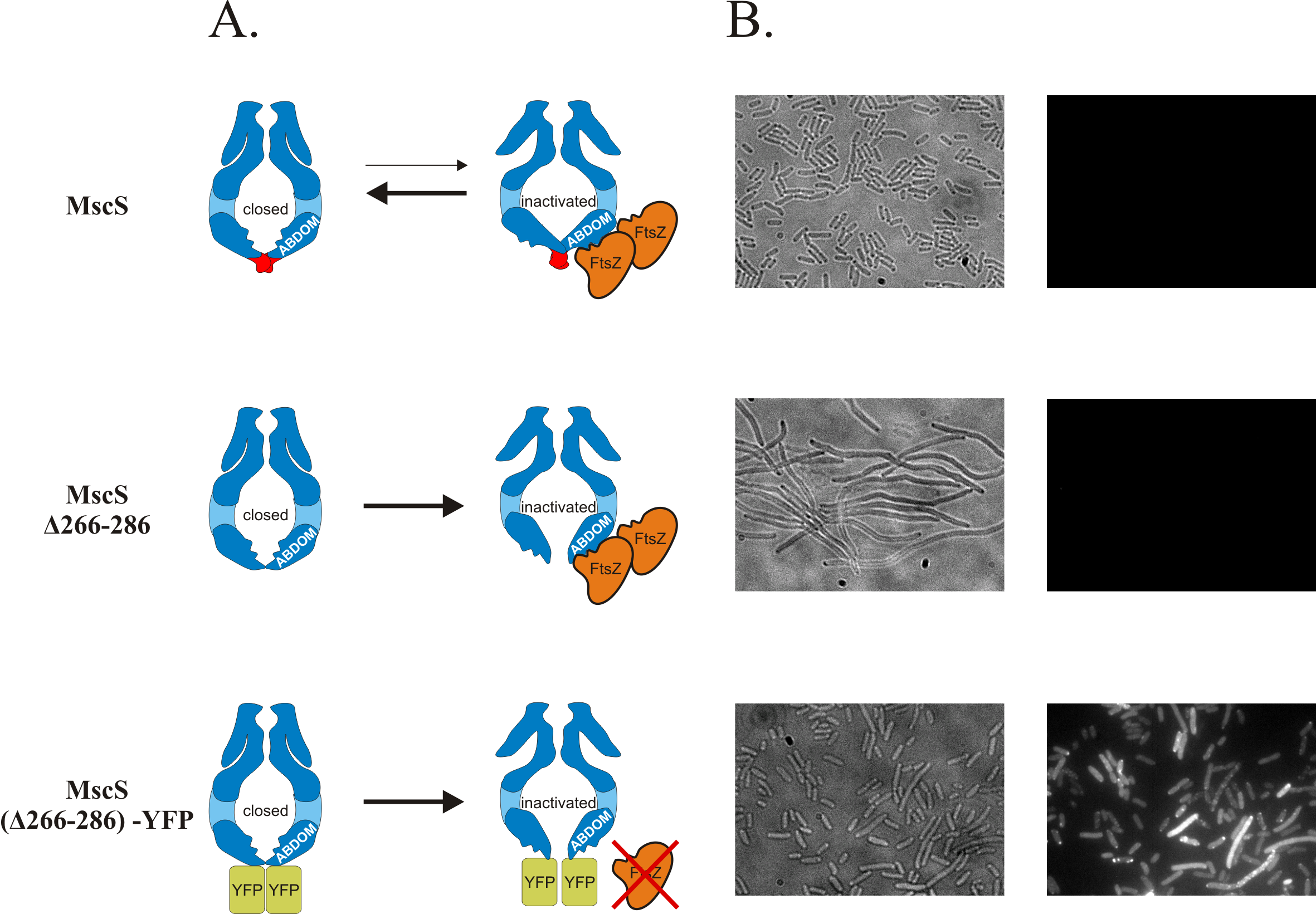

Supplement: S7 Fig — C-terminal YFP is a steric obstacle for the FtsZ binding to MscSΔ266–286. A. Cartoons of conformational changes of MscS, MscSΔ266–286 and MscS-YFP during their closed–to–inactivated transitions (cartoons were drawn according to [17]), and resultant FtsZ binding. Arrows indicate possible kinetic transitions of the channel. In each case the direction of the thick arrow indicates the more probable channel conformation. Under non-stress conditions MscS (upper row) resides in closed state that is noncompetent in FtsZ binding. Under the same conditions MscSΔ266–286 (middle row) resides in a permanent inactivated state, which makes the FtsZ binding possible. We assume that the binding of FtsZ is chronic and it results in cell filamentation. Fusing YFP to C-terminus of MscSΔ266–286 (lower row) prevents FtsZ binding and prevents cell filamentation. B. Microscopic images of cells expressing corresponding constructs (bright field on the left, fluorescence on the right). (TIF) [file pone.0127029.s007.tif]

**S8A Fig.**

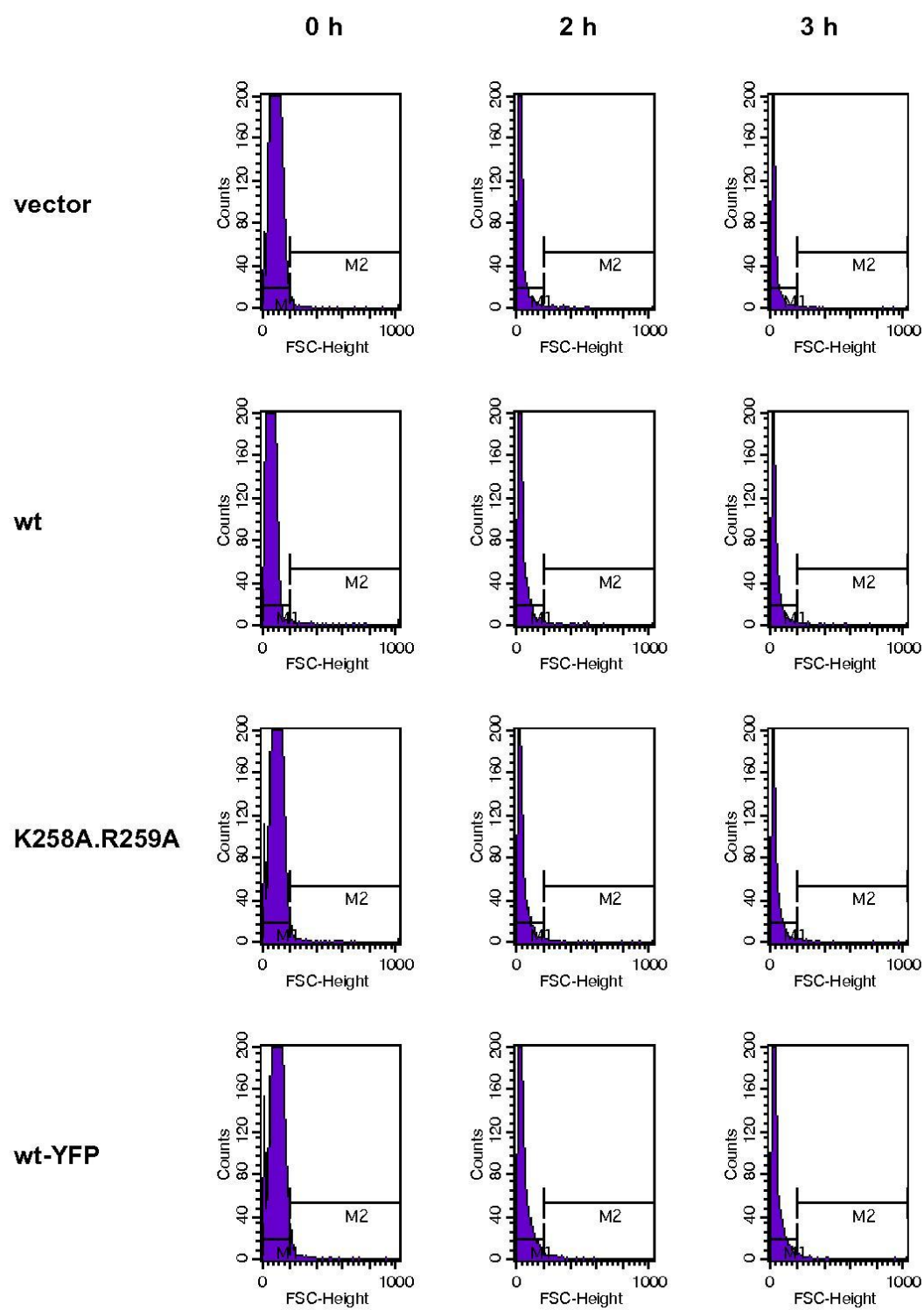

**S8B Fig.**

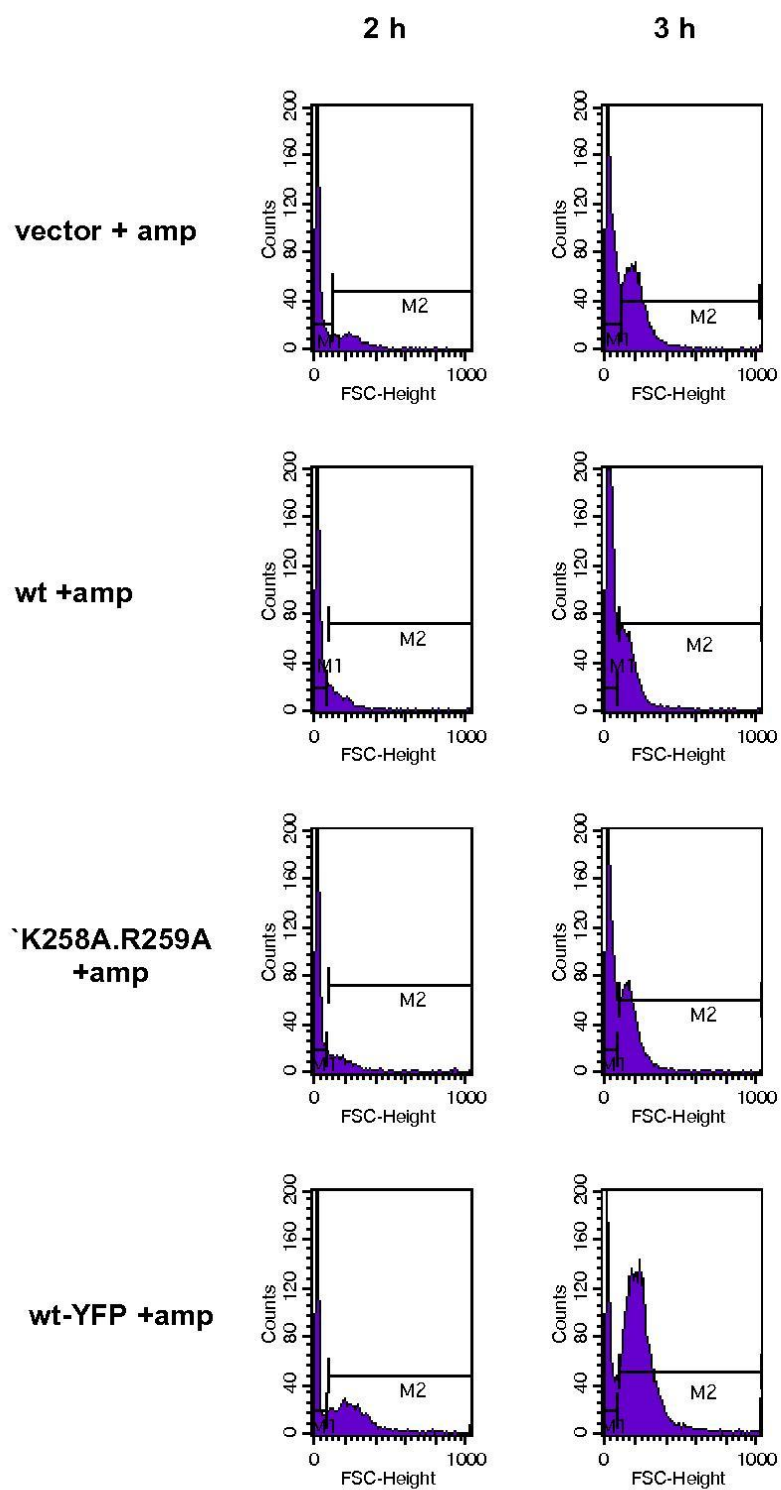

**S8C Fig.**

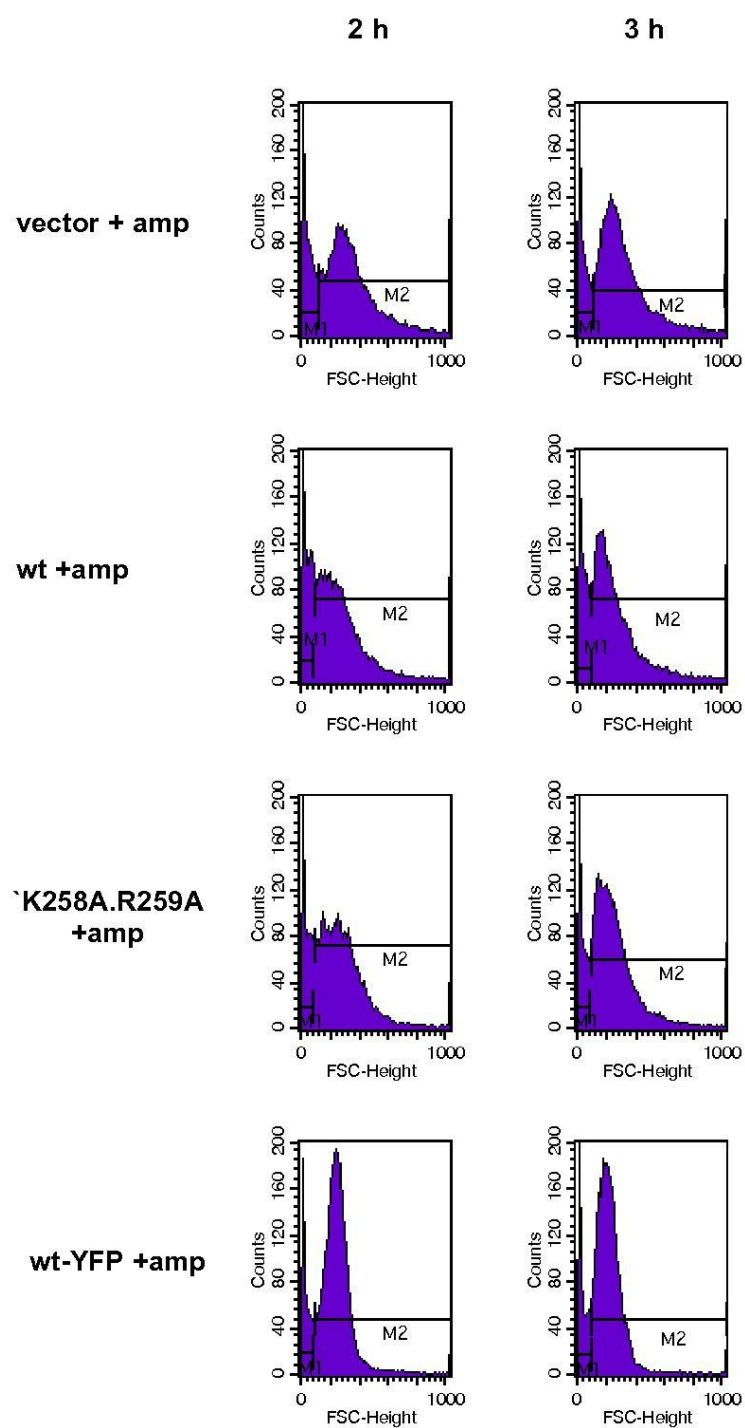

**S8D Fig.**

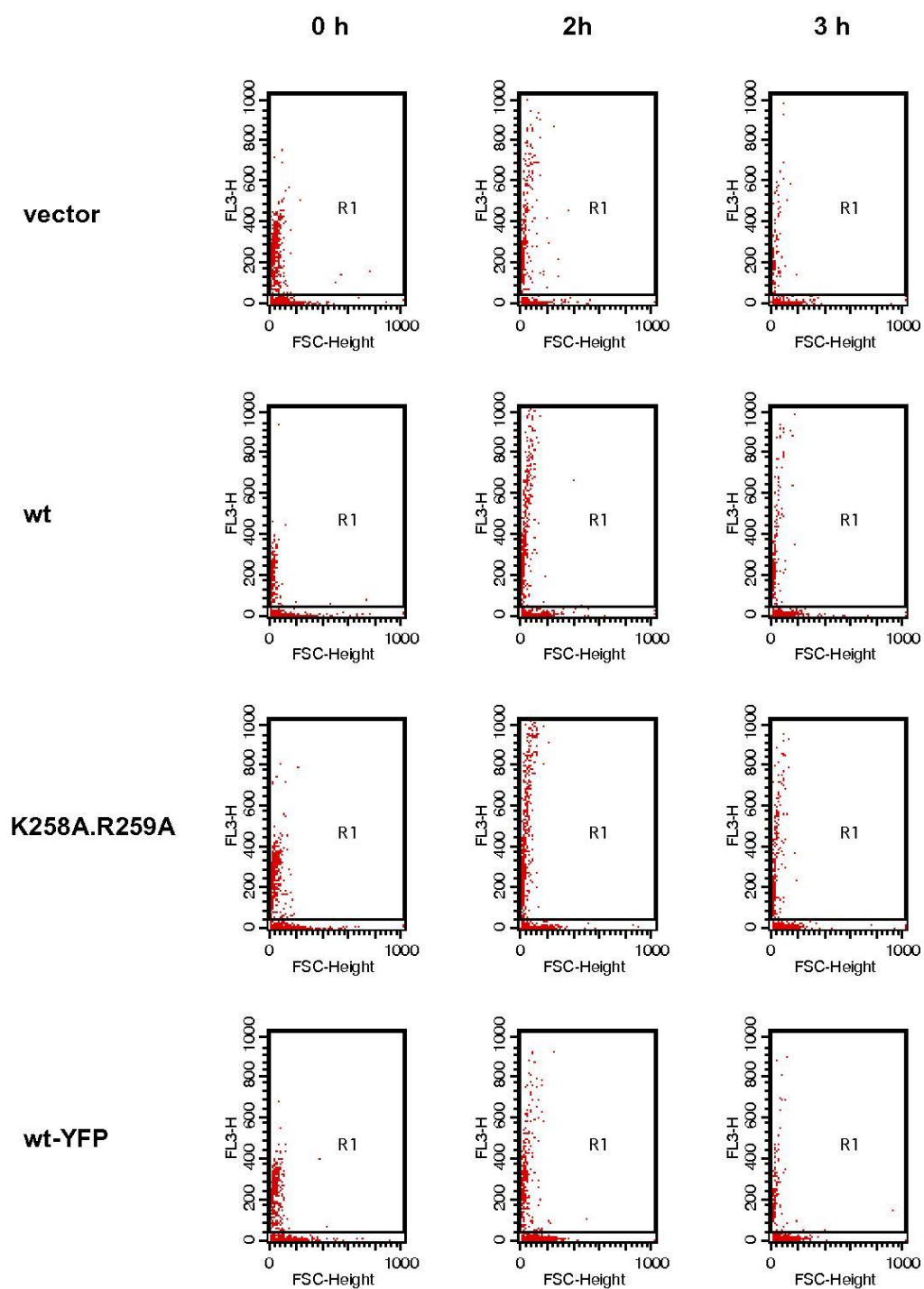

**S8E Fig.**

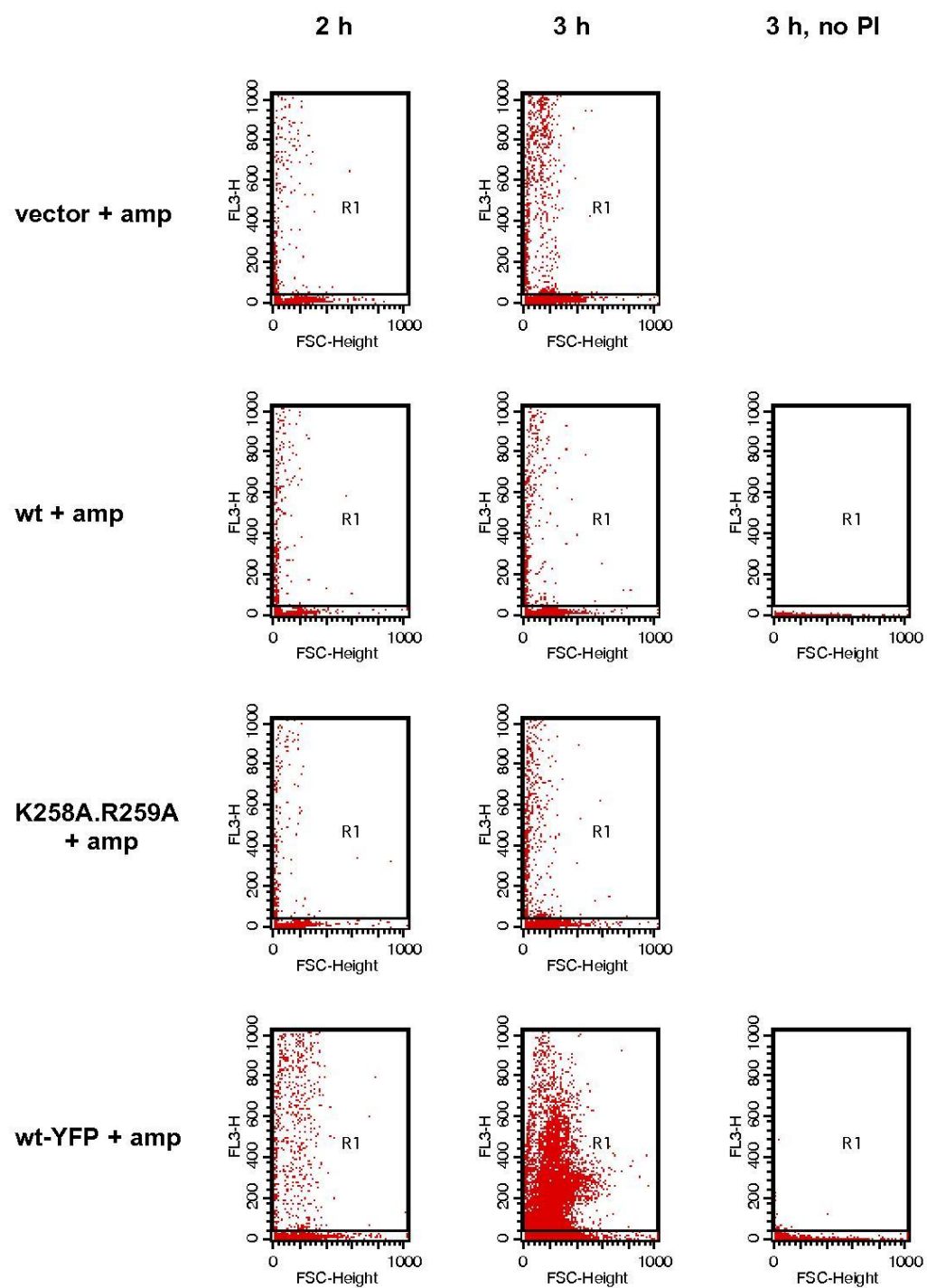

**S8F Fig.**

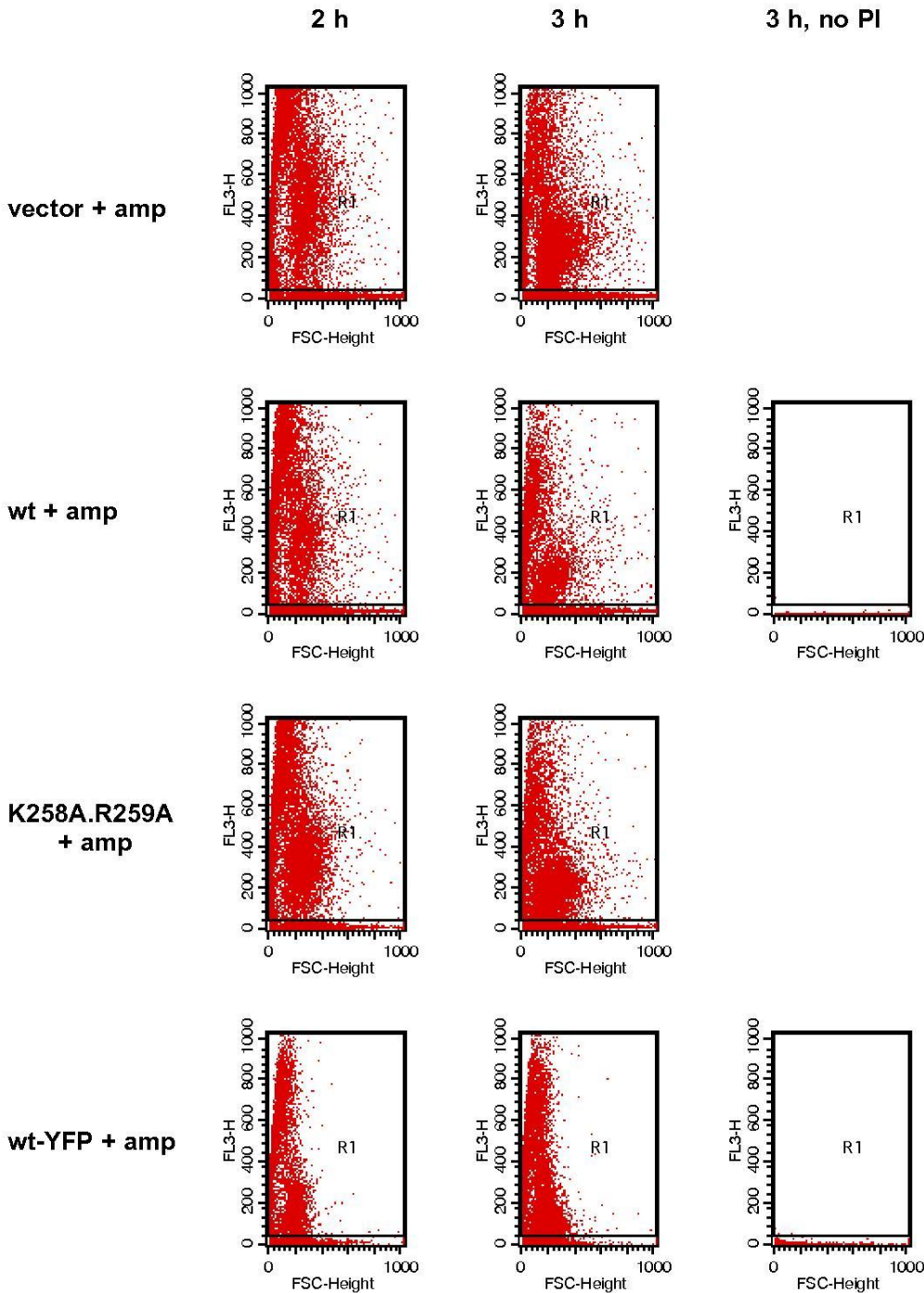

Supplement: S8 Fig — In A, B, C forward scatter histograms are displayed. M1 and M2 ranges refer to two populations of cells short and long ones, respectively. Under control conditions (A.) only short cells were observed (M1 range). In the presence of ampicillin (B., C.) longer cells were observed additionally (M2 range). Lowest number of longer cells was observed in wt MscS. The borderline between M1 and M2 range was set manually to assign >90% of control cells to M1. In D, E, F fluorescence scatter plots are displayed. Damaged cells were stained with propidium iodide (PI). Cells with fluorescence above the background were counted as damaged cells (R1 area). The lowest number of damaged cells was observed in wt MscS. The level of fluorescence of unstained cells (horizontal line in each sample) was manually set as a fluorescence background (as seen in samples presented in E, F rightmost column) and was kept constant for all samples. Cells were grown in LB alone (A, D), and in LB with low (1.6 μg/ml; B, E) or high (4.1 μg/ml; C, F) concentration of ampicillin. All samples presented in A, B, C, D, E, (PDF) [file pone.0127029.s008.pdf]

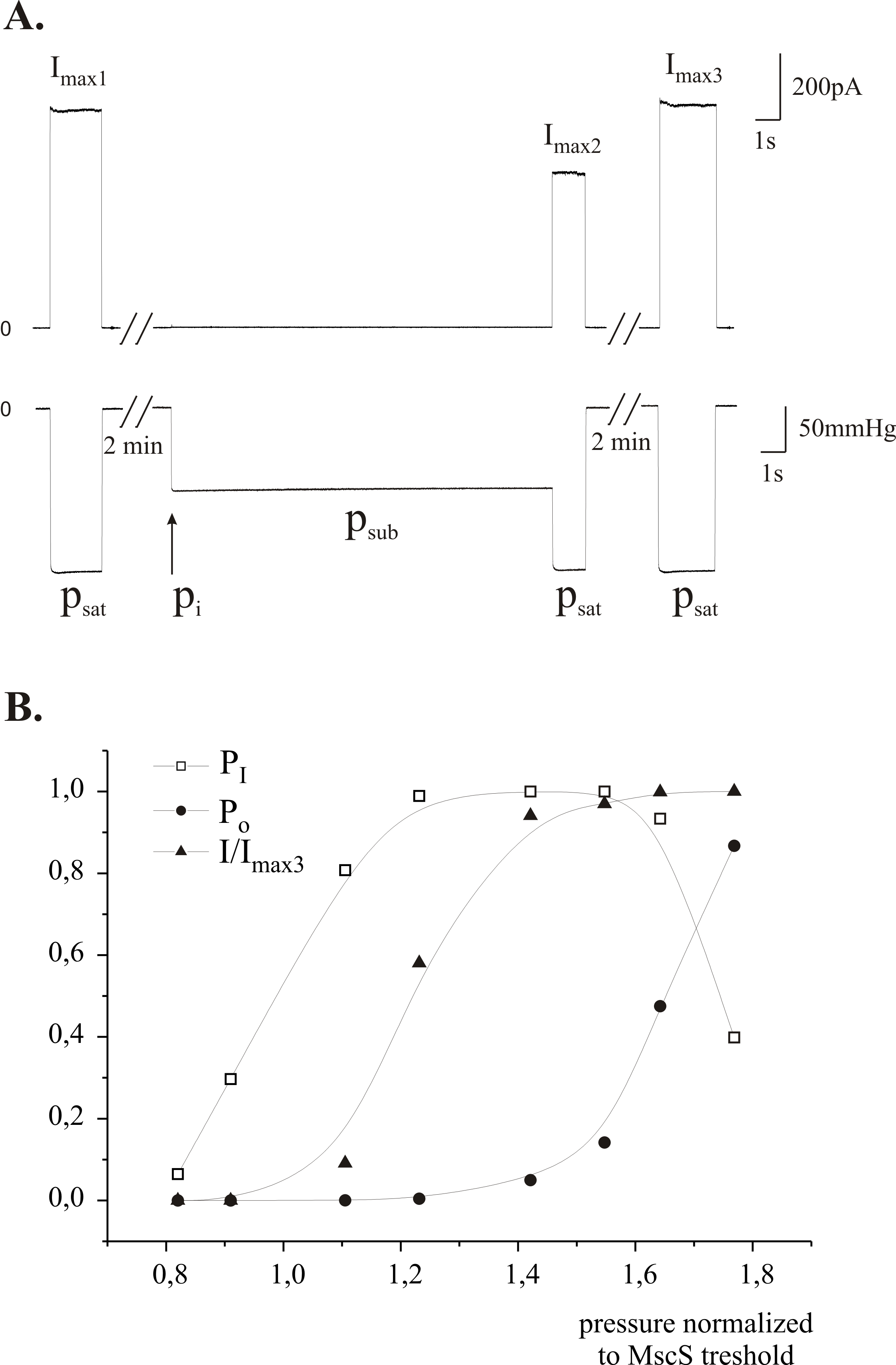

Supplement: S9 Fig — A. Experimental protocol: 1) Primary test: application of saturating pressure (psat) results in Imax1, which is a total current of all open MscS channels present in the patch. 2) Main test: after 2 min MscS traces were recorded with various preconditioning 30s-pressure pulses (arrow marks their application). At subthreshold pressures pi = sub, MscS channels do not open, but some of them inactivate and do not open when saturating pressure is applied (Imax2<Imax1). 3) Final test, identical to the primary test: after 2 min at zero pressure the channels recover from the inactivated state and they reopen (Imax3 = Imax1). Then we calculated a probability of inactivation Pi = 1- Imax2/Imax1, which is plotted in (B.) (white squares). Electrophysiological procedures were similar to those described previously. B. The probability of inactivation (Pi), open probability (Po), and current (I) normalized to maximal current (Imax) are shown as functions of pressure normalized to the activation threshold (95 mmHg in this patch). Note that Pi is higher than Po at low applied pressures. The mean single-channel open probability (P o) during the pressure pulse was calculated by integrating the current passing through all active channels (I) during the pulse and dividing this integral by the current through a single open channel (i) and number of active channels (N) according to the formula P o = I/Ni. (TIF) [file pone.0127029.s009.tif]
