# Supplementary material for: Cytoplasmic Domain of MscS Interacts with Cell Division Protein FtsZ: A Possible Non-Channel Function of the Mechanosensitive Channel in Escherichia Coli
Source: PLoS One. 2015 May 21;10(5):e0127029. doi: 10.1371/journal.pone.0127029 (PMC4440785; doi:10.1371/journal.pone.0127029)
Supplement: S1 Table — Peptides identified only in the samples from MBP-ABDOM but not from MBP pull-down are shown. The table lists peptides with scores >75 from both experiments described in Materials and Methods. We selected FtsZ for further experimentation since: i) ClpX interacts with FtsZ suggesting that its binding to ABDOM may be indirect (i.e. mediated through FtsZ), and, moreover, we observed the ABDOM-induced filamentation in clpX - strain (data not shown); ii) we observed the ABDOM-induced filamentation in recA - strain (data not shown); iii) MreB was identified with a low score and interference with its function results in round cells and not in the cell filamentation. Moreover, MreB possibly associates with FtsZ, so that its interaction with ABDOM could be indirect. (DOCX) [file pone.0127029.s010.docx]

**S1 Table**

| Protein | gi number | Peptide | m/z (charge) | score |
| --- | --- | --- | --- | --- |
| FtsZ | 40863 | TAVGQTIQIGSGITK + Oxidation (M) | 737.413 (2) | 120 |
|  |  | MAFAEQGITELSK | 720.857 (2) | 111 |
|  |  | VTVVATGIGMDK + Oxidation (M) | 603.823 (2) | 104 |
|  |  | LDEFETVGNTIR | 697.342 (2) | 83 |
|  |  | VIGVGGGGGNAVEHMVR + Oxidation (M) | 812.912 (2) | 83 |
| ClpX | 62288037 | DVSGEGVQQALLK | 672.364 (2) | 104 |
|  |  | SNILLIGPTGSGK | 628.872 (2) | 83 |
|  |  | NGDTSNGVELGK | 595.791 (2) | 78 |
| OmpC | 1788544 | NGNPSGEGFTSGVTNNGR | 882.903 (2) | 127 |
|  |  | INLLDDNQFTR | 674.848 (2) | 82 |
|  |  | FQDVGSFDYGR | 645.789 (2) | 76 |
| RecA | 16130606 | IGVMFGNPETTTGGNALK + Oxidation (M) | 911.957 (2) | 92 |
|  |  | ALAAALGQIEK | 542.821 (2) | 78 |
| HCP | 7404426 | QTLLGAADTLIDLVSR | 843.479(2) | 92 |
| MreB | 76363877 | NYGSLIGEATAER | 690.839(2) | 101 |
|  |  | VALEQCPPELASDISER | 957.448(2) | 79 |
