# Supplementary material for: Cytoplasmic Domain of MscS Interacts with Cell Division Protein FtsZ: A Possible Non-Channel Function of the Mechanosensitive Channel in Escherichia Coli
Source: PLoS One. 2015 May 21;10(5):e0127029. doi: 10.1371/journal.pone.0127029 (PMC4440785; doi:10.1371/journal.pone.0127029)
Supplement: S2 Table — Experimental data from S8 Fig, panel C were analyzed and percent of elongated cells (M2 range) and median cell scatter is shown for cell treated with 4.1 μg/ml ampicillin. (DOCX) [file pone.0127029.s011.docx]

**S2 Table**

|  | % of elongated cells (M2 range) | Median cell scatter |
| --- | --- | --- |
| vector | 82 | 271 |
| MscS | 77 | 219 |
| MscS-K258A/R259A | 84 | 224 |
| MscS-YFP | 86 | 206 |
